# Explainable AI-Derived Spatial Pathological Features of Tumor, Necrosis, and Lymphocytes Identify Key Histological Signatures for Residual Cancer Burden Assessment in Breast Cancer

**DOI:** 10.3390/diagnostics16172798

**Published:** 2026-08-31

**Authors:** Xin Shu, Fan Wang, Tiancheng Zhao, Yun Zhang, Yao Zhou, Liu Yang, Xiujuan Xiong, Libin Deng

**Affiliations:** 1 School of Public Health, Jiangxi Medical College, Nanchang University, Nanchang 330006, China; 2 Jiangxi Provincial Key Laboratory of Disease Prevention and Public Health, Nanchang University, Nanchang 330006, China; 3 Data Governance Center, Nanchang People’s Hospital, Nanchang 330009, China; 4 David Geffen School of Medicine, University of California, Los Angeles, CA 90095, USA; 5 Department of Pathology, The First Hospital of Nanchang City/The Third Affiliated Hospital of Nanchang University, Nanchang 330006, China; 6 Nanchang People’s Hospital, Nanchang 330009, China; 7 School of Basic Medical Sciences, Jiangxi Medical College, Nanchang University, Nanchang 330006, China

**Keywords:** spatial pathological features, residual cancer burden, breast cancer, explainable artificial intelligence, tumor microenvironment

## Abstract

**Background:** Conventional histopathological assessment of residual cancer burden (RCB) following neoadjuvant therapy for breast cancer relies on manual visual evaluation, lacking objective quantification of spatial interactions among tumor, necrosis, and lymphocyte compartments. This limits prognostic accuracy and impedes personalized treatment stratification. **Methods:** We developed an explainable AI framework to segment three tissue compartments from H&E patches and constructed 11 spatial pathological features. This two-center study included 347 post-neoadjuvant patients (182 development, 165 external validation) plus 843 TCGA-BRCA cases for exploratory external validation. VGG-16, ResNet-50, and ViT-Base were evaluated; multiple instance learning identified target regions for feature construction. Six machine learning models with LASSO-selected features were interpreted via SHAP and Grad-CAM, with TCGA-BRCA serving as an exploratory external cohort. **Results:** VGG-16 achieved optimal segmentation (all AUCs > 0.90). In the external validation set, XGBoost achieved AUCs of 0.781 for Task 1 (RCB-0 vs. RCB-1+2+3) and 0.896 for Task 2 (RCB-0+1 vs. RCB-2+3). For Task 3 (RCB-0+1+2 vs. RCB-3), the model achieved an AUC of 1.000, with complete separation between groups. SHAP analysis revealed that the Necrotic Tumor Mixed Cluster (T+N) was the dominant indicator for Task 1 (mean |SHAP| = 0.439) and Task 2 (mean |SHAP| = 0.803), while the Pure Necrotic-Rich Zone (N-only) was the dominant indicator for Task 3 (mean |SHAP| = 1.967). In exploratory TCGA association analyses, the Pure Tumor-Rich Zone (T-only) exhibited a positive correlation (ρ = 0.706, *p* < 0.001; T-only is a feature used to derive this model-estimated RCB-like score), whereas the Lymphocyte-Infiltrated Necrotic Zone (N+L) showed a negative correlation (ρ = −0.407, *p* < 0.001) with the model-estimated RCB-like score, linking tumor-rich zones to immunosuppression and lymphocyte-infiltrated necrotic zones to immune activation. **Conclusions:** Our spatial pathological features effectively estimate RCB, with T+N and N-only as the predominant SHAP-identified indicators across the three tasks. Exploratory analyses further suggest that tumor-rich and lymphocyte-infiltrated necrotic zones correlate with opposing immune phenotypes, supporting personalized breast cancer management.

## 1. Introduction

Breast cancer remains a preeminent global health challenge, accounting for approximately 2.3 million new cases and 778,000 deaths annually [1,2,3]. Among women aged 40 to 64 years, it has become the primary malignancy contributing to cancer incidence and mortality [2,4]. For patients with locally advanced breast cancer, neoadjuvant therapy (NAT) is the established standard of care, aimed at downstaging primary tumors, improving surgical resectability, and providing a crucial window to assess therapeutic sensitivity [5,6]. In this clinical setting, Residual cancer burden (RCB) has emerged as the most robust prognostic indicator following NAT [7,8]. Calculated by integrating the dimensions of the primary tumor bed with the extent of regional lymph node involvement, the RCB score offers a precise post-treatment risk stratification [9]. Studies have demonstrated that each 1-unit increase in the RCB score is associated with an 82% higher risk of recurrence or metastasis, providing a validated survival evaluation that transcends diverse breast cancer subgroups and clinical centers [10,11].

However, the clinical utility of traditional RCB assessment is inherently limited by its reliance on manual postoperative histopathological evaluation [11]. Manual assessment carries substantial inter-observer variability and imposes heavy workloads on pathologists [11,12]. Currently, only 19% to 60% of patients achieve a pathological complete response (pCR), while a significant proportion experience minimal response or even disease progression during therapy [13]. Furthermore, most research into discriminative biomarkers has historically converged on a binary classification of pCR versus non-pCR status—distinguishing only between the total absence or presence of residual disease [8,13]. This binary reductionism inherently overlooks the biological complexity within the four-category RCB system. For patients with varying degrees of residual disease (RCB-1, 2, and 3), the risk of long-term recurrence varies significantly, yet these nuanced differences are often lost in simplified classification frameworks [5,10]. Consequently, establishing a standardized framework for refined multi-grade RCB evaluation using postoperative resection specimens is essential for identifying patients who may require treatment intensification or de-escalation [14,15].

Despite the potential of deep learning in digital pathology, significant translational bottlenecks persist [16]. First, the “black box” nature of many algorithms hinders clinical acceptance [17]. Clinicians require a transparent understanding of the histological rationale behind a model output to ensure it aligns with established pathological logic [18,19,20,21]. Second, many existing models suffer from limited feature dimensionality, often focusing exclusively on tumor-cell density while neglecting the spatial interaction patterns between tumor, necrosis, and lymphocyte regions [22,23,24]. Pathological evidence increasingly suggests that the spatial architecture of the tumor microenvironment (TME) is a fundamental indicator of therapeutic response [25,26]. These spatial relationships reflect the underlying biological dynamics of tumor regression and immune activation but remain largely underutilized in conventional computational models [27].

Explainable artificial intelligence (XAI) integrated with spatial pathological interpretable features (PIFs) offers a potential framework to address these challenges [19,28,29,30,31]. By applying multiple instance learning and spatial analytical methods, it becomes possible to quantify multi-component histological patterns from whole slide images (WSIs) that are both intuitive to pathologists and computationally consistent [19,29]. Previous digital pathology studies for post-NAT RCB assessment commonly adopt two mainstream strategies [14,32,33]. Some studies extract black-box patch-based deep learning embeddings lacking transparent pathological interpretation [34,35]. Others rely on basic morphological metrics to quantify tissue components in isolation, failing to depict multicompartment spatial interplay within the TME [26,36,37,38]. Currently, research integrating XAI with PIFs to specifically support multi-grade RCB classification remains insufficiently explored [28]. Different from these approaches, our eleven spatial pathological features are derived from segmented mutually exclusive subregions covering residual tumor, necrosis, and lymphocyte-rich areas. Instead of merely computing regional area fractions, this set of spatial features quantitatively characterizes the complex co-localization patterns among multiple tissue components within the TME [26].

In this study, we developed a framework based on spatial PIFs to evaluate RCB grades from postoperative resection specimens after neoadjuvant therapy. We evaluated the analytical performance and applicability of these features across multi-center and The Cancer Genome Atlas Breast Cancer cohort (TCGA-BRCA), further exploring the contribution of key spatial signatures to provide a biologically grounded reference for clinical neoadjuvant treatment decisions.

## 2. Materials and Methods

**Figure 1:**
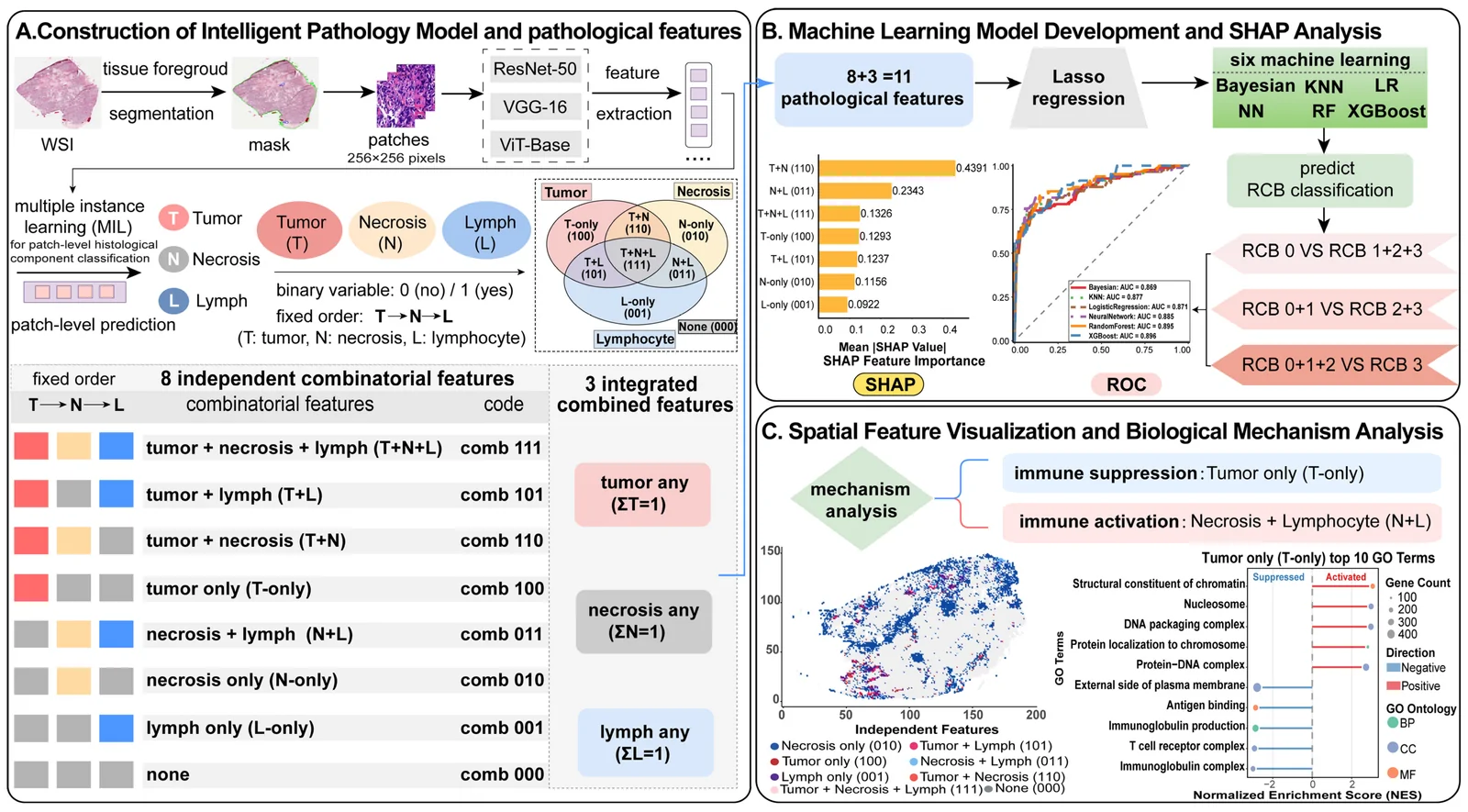
Schematic workflow of the intelligent pathological framework for estimating RCB. (**A**) Image processing and feature extraction. Whole slide images (WSIs) were segmented into patches; three deep learning architectures were evaluated for classifying tumor, necrosis, and lymphocyte regions. Eleven spatial pathological features (eight combinatorial and three global) were subsequently defined. (**B**) Model development and interpretability. LASSO regression was applied for feature selection; six machine learning algorithms were trained for three RCB binary classification tasks. SHAP analysis quantified the contribution of each feature. (**C**) Spatial visualization and biological validation. Key feature distributions were mapped onto WSIs, and GO enrichment analysis was performed to explore underlying molecular mechanisms.

The overall study design consists of three phases: deep learning model construction and derivation of 11 spatial pathological features, machine learning model development and SHAP analysis for RCB classification, and spatial feature visualization and biological mechanism interpretation (Figure 1).

### 2.1. Study Design and Patient Selection

A total of 347 patients with breast cancer who received neoadjuvant therapy were enrolled. Of these, 182 patients from Nanchang People’s Hospital formed the model development cohort, which was stratified and randomly split (8:2) into a training set (*n* = 147) and an internal test set (*n* = 35). Ten-fold cross-validation was applied for model construction and parameter optimization. An independent external validation cohort consisted of 165 patients from Hubei Provincial Hospital of Integrated Traditional Chinese and Western Medicine. Additionally, 843 breast cancer cases with available pathological images, clinical data, and omics data were selected from the TCGA-BRCA database as an exploratory external cohort. All WSIs were digitized from H&E sections of surgical resection specimens after completion of neoadjuvant therapy, the same tissues used for clinical RCB scoring.

The inclusion criteria were primary invasive breast cancer, receipt of standardized neoadjuvant therapy, availability of histological slides, and complete clinical RCB evaluation data. The exclusion criteria were concurrent other systemic malignancies, special breast cancer histological subtypes, pure carcinoma in situ, and recurrent breast cancer.

### 2.2. Pathological Assessment and Outcome Measures

The primary outcome measure in this study was the RCB system, which was derived from quantitative pathological measurements to assess residual tumor burden [33]. Prespecified cutoffs defined four categories: RCB-0 (score 0, pCR), RCB-1 (>0 to 1.36, minimal residual disease), RCB-2 (1.37–3.28, moderate residual disease), and RCB-3 (>3.28, extensive residual disease) [10,39].

Immunohistochemistry was used to assess estrogen receptor (ER), progesterone receptor (PR), androgen receptor (AR), and human epidermal growth factor receptor 2 (HER2) status. The Ki-67 index was dichotomized using a cutoff of 20% [5]. Specimens from the external validation cohort were centrally re-evaluated before inclusion, and all postoperative specimens were independently reviewed by two senior pathologists.

### 2.3. Image Processing and Deep Learning Framework

WSIs were acquired using UNIC PRECICE 610 × 1 (Nanchang People’s Hospital) and KFBio KF-PRO-005 scanners (Hubei Provincial Hospital of Integrated Traditional Chinese and Western Medicine). Standardized H&E staining was performed at both centers. Scanning at 20× and 40× magnification provided resolutions of 0.5 μm per pixel and 0.25 μm per pixel, respectively. Valid tissue areas were extracted as 256 × 256 pixel patches (20×) and 512 × 512 pixel patches (40×). All patches were processed with channel-wise RGB normalization before feature extraction to mitigate inter-center heterogeneity, scanner-associated bias, and color variations from H&E staining.

Following preprocessing procedures, we adopted Visual Geometry Group network-16 (VGG-16), Residual Network with 50 layers (ResNet-50), and Vision Transformer Base (ViT-Base) as backbone feature extractors. Subsequently, a clustering-based attention multiple instance learning (CLAM) framework [16] was implemented to classify patches into three histological components: tumor (T), necrosis (N), and lymphocytes (L). All WSIs analyses were performed on a workstation running Windows 11 professional for workstations, equipped with an Intel(R) Xeon(R) Gold 6338 CPU, 512 GB RAM, an NVIDIA A100-SXM4 GPU (80 GB VRAM), and an 18 TB HDD for data storage. On average, feature extraction for each WSI takes approximately 200 s, and downstream model inference requires around 20 s. The peak GPU memory consumption during processing is approximately 20 GB, and CPU memory usage remains far below the 512 GB system limit.

### 2.4. Definition of Digital Pathological Features

To systematically characterize the spatial heterogeneity of the tumor microenvironment following NAT, we developed a structured framework comprising 11 digital pathological features. Unlike traditional single-component assessments, the digital pathological features framework integrates the binary status (1 = presence, 0 = absence) of tumor (T), necrosis (N), and lymphocytes (L) into a fixed T-N-L logical sequence, enabling a multi-dimensional profiling of the post-treatment residual landscape (as shown in Figure 1). This approach defines eight independent spatial patterns to delineate mutually exclusive pathological niches (e.g., Pure Tumor-Rich Zone (T-only) and Lymphocyte-Infiltrated Necrotic Zone (N+L)). By characterizing these spatial co-occurrence patterns, the framework effectively captures localized microenvironmental interactions including the immune-necrotic interface, which are frequently neglected in conventional pathological scoring systems. Furthermore, we derived three integrated global indices (e.g., Global Tumor Compartment (T-any)) to quantify the overall regional burden of individual components, ensuring good compatibility with routine clinical pathological assessment. Each digital pathological feature was quantified as the proportional abundance of patches within the qualified tissue regions of individual WSIs, serving as computable pathological biomarkers for the precise assessment of therapeutic response.

### 2.5. Machine Learning Model Construction and Validation

Six machine learning algorithms, including Logistic Regression (LR), K-Nearest Neighbor (KNN), Random Forest (RF), eXtreme Gradient Boosting (XGBoost), Bayesian, and Neural Network (NN), were developed using eight combinatorial features derived from the original eleven features by excluding the three global indices, with comb 000 further excluded from model inputs to avoid exact collinearity since the eight proportions sum to one.

To address the clinical spectrum of treatment response, we formulated three sequential binary classification tasks reflecting the increasing residual disease severity (RCB 0 vs. 1+2+3; RCB 0+1 vs. 2+3; RCB 0+1+2 vs. 3). Feature selection was conducted via LASSO regression with 10-fold cross-validation. Model performance was evaluated using the area under the receiver operating characteristic (ROC) curve (AUC), accuracy, F1-score, precision, sensitivity, specificity, and calibration. Calibration was assessed using calibration curves. Continuous RCB scores were further analyzed via regression analysis to explore linear and nonlinear correlations with digital pathological features.

### 2.6. Interpretability and Biological Exploration

Two complementary interpretability approaches combining Shapley Additive Explanations (SHAP) analysis and Gradient-weighted Class Activation Mapping (Grad-CAM) visualization were adopted to interpret model behaviour from the perspectives of global feature contribution and local spatial attention. For biological validation, the TCGA-BRCA cohort was stratified according to the median value of core digital pathological features. Differentially expressed genes were screened, followed by GO functional annotation, KEGG pathway enrichment, and GSEA analyses to reveal potential biological pathways mediated by TME spatial patterns.

### 2.7. Statistical Analysis

Analyses were performed using Python (v3.9.12) and R (v4.3.2). Continuous variables were compared using the Wilcoxon rank-sum test. Spearman’s correlation analysis was used to assess the association between digital pathological features proportions and RCB scores. Statistical significance was defined as two-tailed *p* < 0.05.

## 3. Results

### 3.1. Baseline Characteristics

A total of 347 patients with breast cancer who received neoadjuvant therapy were enrolled in this study, including 182 patients in the modeling cohort from Nanchang People’s Hospital and 165 patients in the external validation cohort from Hubei Provincial Hospital of Integrated Traditional Chinese and Western Medicine. The modeling cohort was randomly divided 8:2 into a training set (*n* = 147) and an internal test set (*n* = 35), and the external validation set (*n* = 165) was used to independently evaluate model generalizability. The baseline clinicopathological characteristics of the three cohorts are summarized in Table 1, including age, tumor laterality, RCB class, Ki-67 proliferation index, and ER, PR, AR, and HER2 status. The results demonstrated balanced distributions of key clinical characteristics among cohorts, indicating that the study cohorts were comparable.

### 3.2. Evaluation of Three Deep Learning Models for Identifying Tumor, Necrosis, and Lymphocyte Regions in Histopathology

**Figure 2:**
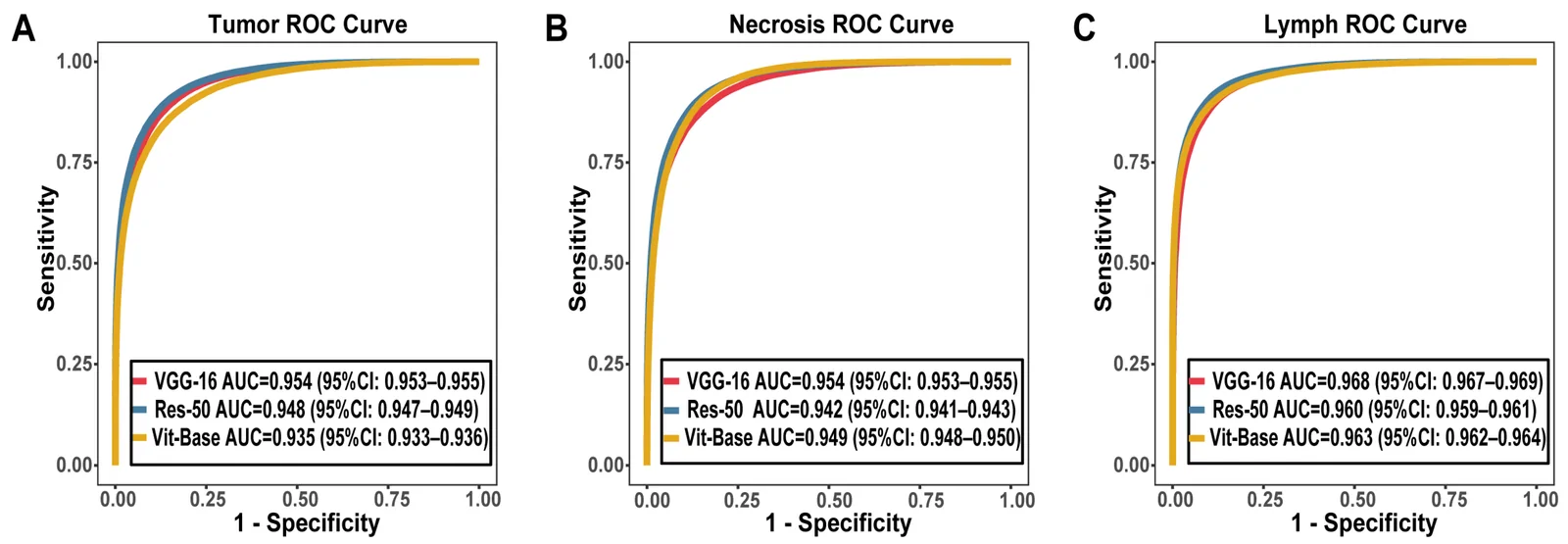
Performance of three deep learning models in identifying core pathological regions. (**A**) ROC curve for tumor region identification. (**B**) ROC curve for necrosis region identification. (**C**) ROC curve for lymphocyte region identification.

We first evaluated three deep learning models, VGG-16, ResNet-50, and ViT-Base, for segmenting tumor, necrosis, and lymphocyte regions on H&E patches. For tumor region identification (Figure 2A), VGG-16 achieved the highest AUC of 0.954 (95% CI: 0.953 to 0.955), followed by ResNet-50 (AUC: 0.948, 95% CI: 0.947 to 0.949) and ViT-Base (AUC: 0.935, 95% CI: 0.933 to 0.936). For necrosis region identification (Figure 2B), VGG-16 remained the optimal performer (AUC: 0.954, 95% CI: 0.953 to 0.955), compared with ViT-Base (AUC: 0.949, 95% CI: 0.948 to 0.950) and ResNet-50 (AUC: 0.942, 95% CI: 0.941 to 0.943). For lymphocyte region identification (Figure 2C), VGG-16 again showed the highest performance (AUC: 0.968, 95% CI: 0.967 to 0.969), followed by ViT-Base (AUC: 0.963, 95% CI: 0.962 to 0.964) and ResNet-50 (AUC: 0.960, 95% CI: 0.959 to 0.961).

Detailed performance metrics for all models are provided in Supplementary Figure S1A–I. Among them, VGG-16 performed best in identifying tumor, necrosis, and lymphocyte regions, providing a stable and accurate basis for generating the 11 subsequent pathological features.

### 3.3. Generation and Interpretable Visualization of 11 Pathological Features

**Figure 3:**
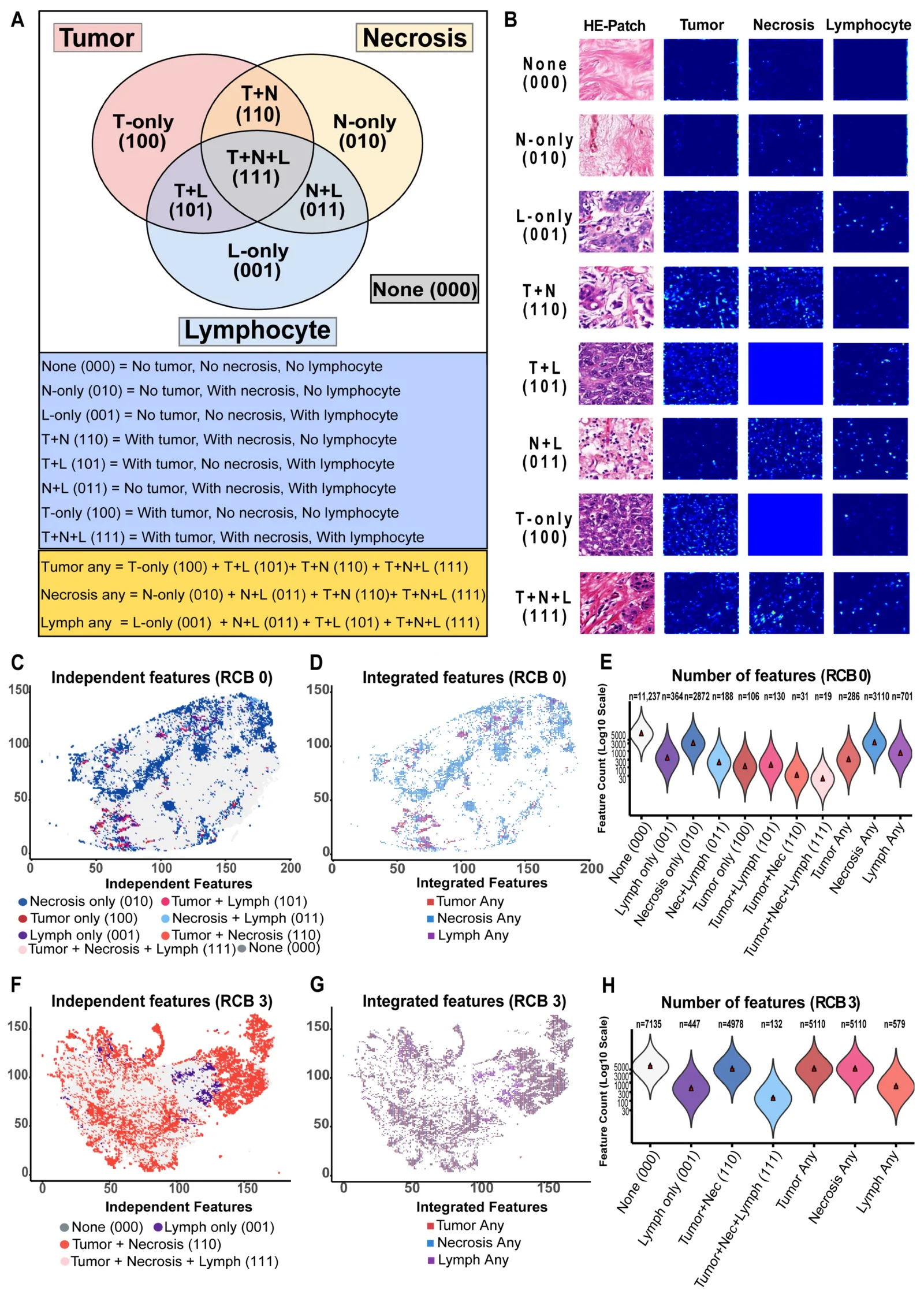
Generation of 11 pathological features and their interpretable visualization. (**A**) The 11 pathological features (8 independent combinatorial features and 3 integrated combined features). (**B**) Grad-CAM spatial visualization results for the 8 independent combinatorial features. (**C**) Spatial distribution heatmap of pathological features in the RCB 0 group. (**D**) Integrated spatial distribution heatmap of integrated combined features in the RCB 0 group. (**E**) Feature count violin plot of pathological features in the RCB 0 group. (**F**) Spatial distribution heatmap of pathological features in the RCB 3 group. (**G**) Integrated spatial distribution heatmap of integrated combined features in the RCB 3 group. (**H**) Feature count violin plot of pathological features in the RCB 3 group.

Based on VGG-16 segmentation, we constructed 11 spatial pathological features, comprising 8 mutually exclusive local spatial patterns and 3 global indices (Figure 3A, Table 2). All features were strictly defined by the co-occurrence of tumor parenchyma, necrosis, and lymphocytes. The core morphological zones included Pure Tumor-Rich Zone (T-only) and Lymphocyte-Infiltrated Necrotic Zone (N+L), while Triple-Coexistence Zone (T+N+L) represented regions with all three components co-existing. The three global indices (T-any, N-any, L-any) quantified the total area proportions of each tissue component across the entire section.

Grad-CAM visualization (Figure 3B) validated the pathological interpretability of the segmentation model, showing high concordance between model attention regions and actual H&E tissue structures. RCB-0 and RCB-3 subgroups displayed opposing spatial organization patterns (Figure 3C–H), with the former dominated by non-tumor components such as Pure Necrotic-Rich Zone (N-only) and Pure Lymphoid-Rich Zone (L-only), and the latter dominated by tumor components such as Necrotic Tumor Mixed Cluster (T+N).

### 3.4. Discriminative Performance of Six Machine Learning Models

**Figure 4:**
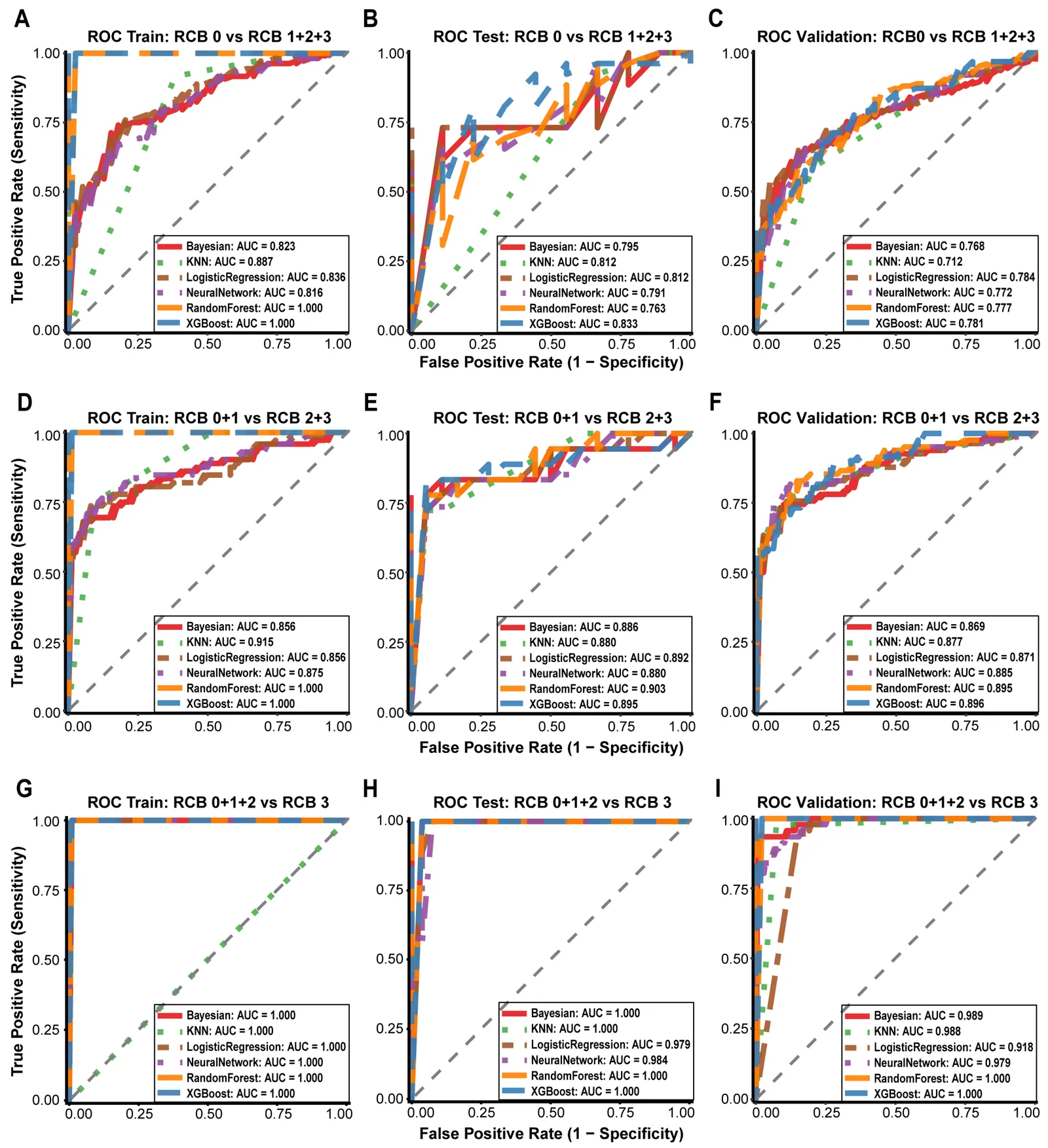
ROC curves of six ML models (Bayesian, KNN, logistic regression, NN, RF, and XGBoost) across different datasets and three binary RCB stratification tasks. (**A**) ROC curves of six ML models in the training set for Task 1 (RCB 0 vs. RCB 1+2+3). (**B**) ROC curves of six ML models in the test set for Task 1 (RCB 0 vs. RCB 1+2+3). (**C**) ROC curves of six ML models in the external validation set for Task 1 (RCB 0 vs. RCB 1+2+3). (**D**) ROC curves of six ML models in the training set for Task 2 (RCB 0+1 vs. RCB 2+3). (**E**) ROC curves of six ML models in the test set for Task 2 (RCB 0+1 vs. RCB 2+3). (**F**) ROC curves of six ML models in the external validation set for Task 2 (RCB 0+1 vs. RCB 2+3). (**G**) ROC curves of six ML models in the training set for Task 3 (RCB 0+1+2 vs. RCB 3). (**H**) ROC curves of six ML models in the test set for Task 3 (RCB 0+1+2 vs. RCB 3). (**I**) ROC curves of six ML models in the external validation set for Task 3 (RCB 0+1+2 vs. RCB 3).

To identify the most discriminative signatures, LASSO regression was applied to eight combinatorial features obtained by removing the three global indices from the original eleven features, with comb 000 excluded from the feature set to avoid exact collinearity (Supplementary Figure S2A–F). We then evaluated six ML algorithms across three binary RCB stratification tasks using training, test, and external validation sets (Figure 4A–I). In Task 1 (RCB-0 vs. 1+2+3), tree-based models (XGBoost and RF) displayed high fitting capacity on the training data. Regarding generalizability, Logistic Regression exhibited the most stable performance in the external validation set (AUC = 0.784), followed by XGBoost (AUC = 0.781) and Bayesian (AUC = 0.768) (Figure 4C). For Task 2 (RCB-0+1 vs. 2+3), discriminative performance improved across all models, with XGBoost and RF yielding AUCs of 0.896 and 0.895 in the external validation set, respectively (Figure 4F). Notably, in Task 3 (RCB-0+1+2 vs. 3), all models achieved consistent high-level discrimination across datasets. In the external validation set, XGBoost and RF reached an AUC of 1.000, while Bayesian (AUC = 0.989) and KNN (AUC = 0.988) yielded comparable results (Figure 4I). This exceptionally high degree of separability is likely largely related to the inherent histopathological contrast between the RCB-3 (*n* = 41 in the external validation set) group, characterized by massive residual tumor beds, and the RCB-0+1+2 group, characterized by absent or minimal residual tumor. Radar charts (Supplementary Figure S3) confirmed that XGBoost maintained balanced accuracy and specificity across these stratification schemes. For continuous RCB score assessment, we assessed six regression models (Supplementary Figure S4). Random Forest (RF) showed the highest coefficient of determination in the external validation set (R^2^ = 0.715, *p* < 0.001), followed by XGBoost (R^2^ = 0.700, *p* < 0.001). The AUC values and their 95% confidence intervals (95% CIs) for all six models across the three RCB classification tasks are summarized in Supplementary Table S1.

### 3.5. Interpretability Analysis: Deciphering Clinical Logic via SHAP

**Figure 5:**
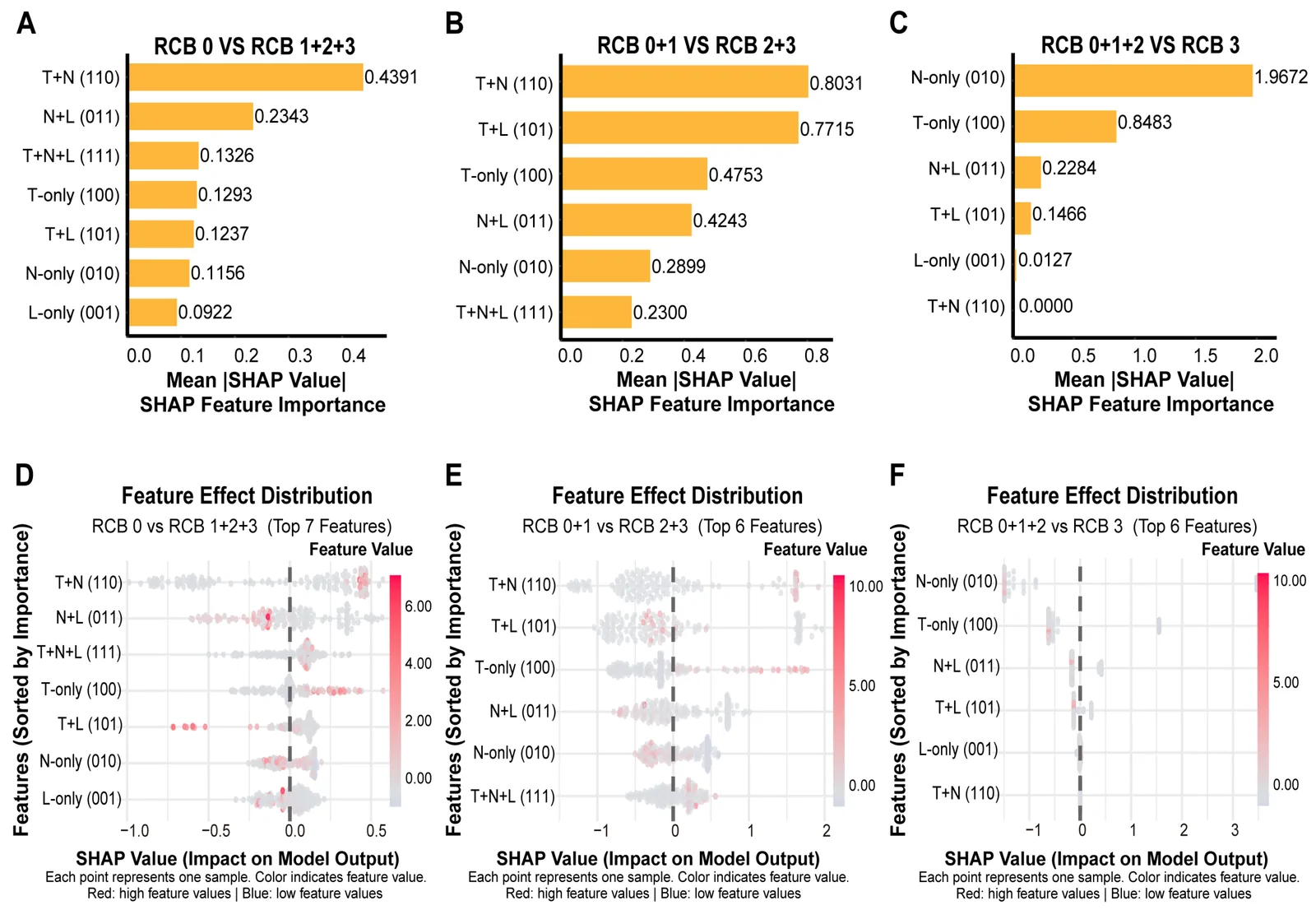
SHAP feature importance and effect distribution of XGBoost across three binary RCB stratification tasks. (**A**) SHAP feature importance bar plot for Task 1 (RCB 0 vs. RCB 1+2+3). (**B**) SHAP feature importance bar plot for Task 2 (RCB 0+1 vs. RCB 2+3). (**C**) SHAP feature importance bar plot for Task 3 (RCB 0+1+2 vs. RCB 3). (**D**) SHAP effect distribution beeswarm plot for Task 1 (RCB 0 vs. RCB 1+2+3). (**E**) SHAP effect distribution beeswarm plot for Task 2 (RCB 0+1 vs. RCB 2+3). (**F**) SHAP effect distribution beeswarm plot for Task 3 (RCB 0+1+2 vs. RCB 3). Note: Bar plots display mean |SHAP| values, reflecting the overall contribution of each feature to model assessment. In beeswarm plots, each dot represents one sample, with red indicating high feature values and gray indicating low feature values.

Considering the compositional nature, we excluded the three global indices and retained the eight combinatorial features (comb 000–comb 111) for LASSO and SHAP analyses. Since the eight proportions sum to one, comb 000 was not retained as a model input to avoid exact collinearity. To elucidate the model’s decision-making logic, SHAP analysis was used to quantify the contribution of each LASSO-selected feature across the three RCB tasks (Figure 5).

In Task 1 (RCB-0 vs. 1+2+3), Necrotic Tumor Mixed Cluster (T+N) emerged as the dominant driving feature (mean |SHAP| = 0.4391), with high feature values directing assessments toward non-pCR (Figure 5A,D). Lymphocyte-Infiltrated Necrotic Zone (N+L) functioned as the primary protective signal for pCR (mean |SHAP| = 0.2343), while Pure Tumor-Rich Zone (T-only) showed a risk-promoting contribution (mean |SHAP| = 0.1293) (Supplementary Figure S5A–G).

In Task 2 (RCB-0+1 vs. 2+3), Necrotic Tumor Mixed Cluster (T+N) remained the primary driver (mean |SHAP| = 0.8031), steering assessments toward high residual burden (RCB-2+3) (Figure 5B,E). Tumor–Lymphocyte Coexistence (T+L) also contributed substantially to risk assessment (mean |SHAP| = 0.7715). Pure Tumor-Rich Zone (T-only) consistently acted as a risk factor (mean |SHAP| = 0.4753), whereas Lymphocyte-Infiltrated Necrotic Zone (N+L) exhibited a protective tendency (mean |SHAP| = 0.4243) (Supplementary Figure S6A–F).

In Task 3 (RCB-0+1+2 vs. 3), Pure Necrotic-Rich Zone (N-only) (mean |SHAP| = 1.9672) and Pure Tumor-Rich Zone (T-only) (mean |SHAP| = 0.8483) were the most powerful indicators for RCB-3 (Figure 5C,F). Notably, extremely low values of these features corresponded to peak SHAP values, strongly driving assessments toward RCB-3 (Supplementary Figure S7A–F). The raw distribution of the Pure Necrotic-Rich Zone (N-only) feature stratified by RCB subgroups is shown in Supplementary Figure S8A. To further evaluate the reliability of the models, we generated calibration curves for the training, internal test, and external validation sets, which are presented in Supplementary Figure S9A–I. Notably, calibration curves for Task 3 were affected by complete separation.

Across all three tasks, the opposing directional effects of tumor-dominant features (risk-promoting) and lymphocyte-associated features (protective) were consistently observed, confirming the biological coherence of the model’s decision-making process.

### 3.6. Exploratory Association Between Spatial Pathological Features and Model-Estimated RCB-like Scores in the TCGA-BRCA Cohort

**Figure 6:**
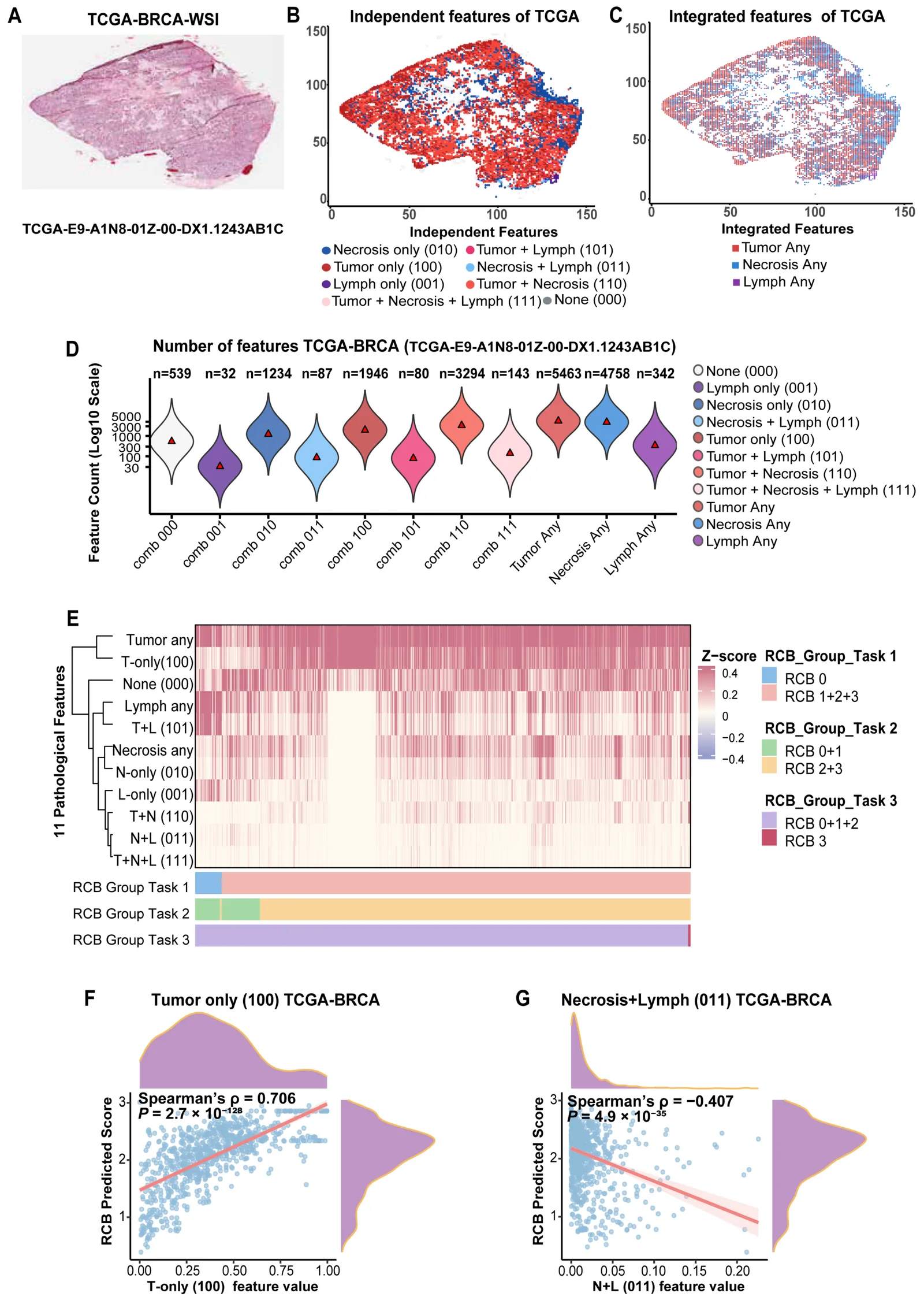
Spatial distribution of pathological features and their correlation with model-estimated RCB-like scores in the TCGA-BRCA cohort. (**A**) Original image of one representative WSI from the TCGA-BRCA cohort. (**B**) Spatial distribution of 8 independent pathological features in this WSI. (**C**) Spatial distribution of 3 integrated pathological features in this WSI. (**D**) Violin plot of log_10_-transformed count distribution of 11 pathological features in this WSI. (**E**) Heatmap of 11 raw pathological features ordered by model-estimated RCB-like scores in the TCGA-BRCA cohort. (**F**) Scatter plot of the correlation between comb 100 (Pure Tumor-Rich Zone (Tumor-only)) and model-estimated RCB-like scores. (**G**) Scatter plot of the correlation between comb 011 (necrosis + lymph, Lymphocyte-Infiltrated Necrotic Zone (N+L)) and model-estimated RCB-like scores. Note: The 11 features visualized in heatmap (**E**) are raw phenotypic measurements directly quantified from WSIs and were not used as model inputs. The model-estimated RCB-like scores used for sample grouping were generated by the optimal machine-learning model trained on eight combinatorial features.

Based on the 11 previously constructed pathological features, we selected one representative WSI from the TCGA-BRCA cohort for visualization and explored correlations between pathological features and model-estimated RCB-like scores (generated by the optimal model trained on eight combinatorial features), using data from all 843 patients in the cohort. Mapping patch-level feature coordinates identified by VGG-16 back to this representative WSI intuitively presented the spatial distribution of each pathological component (Figure 6A–C), showing significant spatial clustering of different pathological features and reflecting the high histological heterogeneity of breast cancer. Violin plots (Figure 6D) showed that, after log10 transformation, the counts of the 11 pathological features differed significantly in this representative WSI. Additionally, Spearman correlation analysis revealed no consistent associations between the 11 pathological features and molecular subtypes in our internal cohort (*N* = 182), except for a few weak correlations (Supplementary Figure S8B). We further conducted HER2-stratified analyses according to HER2 status in this internal cohort (*N* = 182). There were no significant differences in the 11 pathological features between the HER2-any staining and HER2-IHC 0 subgroups (all *p* > 0.05, Supplementary Figure S8C). In the HER2-any staining subgroup (*n* = 169), nine pathological features differed significantly across RCB grades (*p* < 0.05, Supplementary Figure S8D); the limited sample size of the HER2-IHC 0 subgroup (*n* = 13) precluded reliable RCB-stratified statistical testing.

We then applied the optimal machine learning model to the pathological features of all 843 patients in to generate model-estimated RCB-like scores and stratification groups for the three binary tasks. Heatmap analysis (Figure 6E) revealed distinct standardized expression patterns: tumor-centric features, such as Pure Tumor-Rich Zone (T-only) and Global Tumor Compartment (T-any), were predominantly enriched in high estimated RCB groups. Conversely, lymphocyte-centric features, including Pure Lymphoid-Rich Zone (L-only) and Lymphocyte-Infiltrated Necrotic Zone (N+L), tended to cluster in low estimated RCB groups, suggesting a preliminary association between these spatial signatures and residual burden.

Pure Tumor-Rich Zone (T-only) exhibited the strongest positive correlation with the model-estimated RCB-like scores (ρ = 0.706, *p* = 2.7 × 10^−128^). Although Pure Lymphoid-Rich Zone (L-only) showed the strongest inverse correlation (ρ = −0.616, *p* = 4.6 × 10^−89^), we selected Lymphocyte-Infiltrated Necrotic Zone (N+L) (ρ = −0.407, *p* = 4.9 × 10^−35^) as the negative correlate because its mixed necrosis-lymphocyte composition provides the most direct pathological counterpart to T-only (Supplementary Table S2).

### 3.7. Exploratory Analysis of Key Pathological Features and the Tumor Immune Microenvironment in Breast Cancer

**Figure 7:**
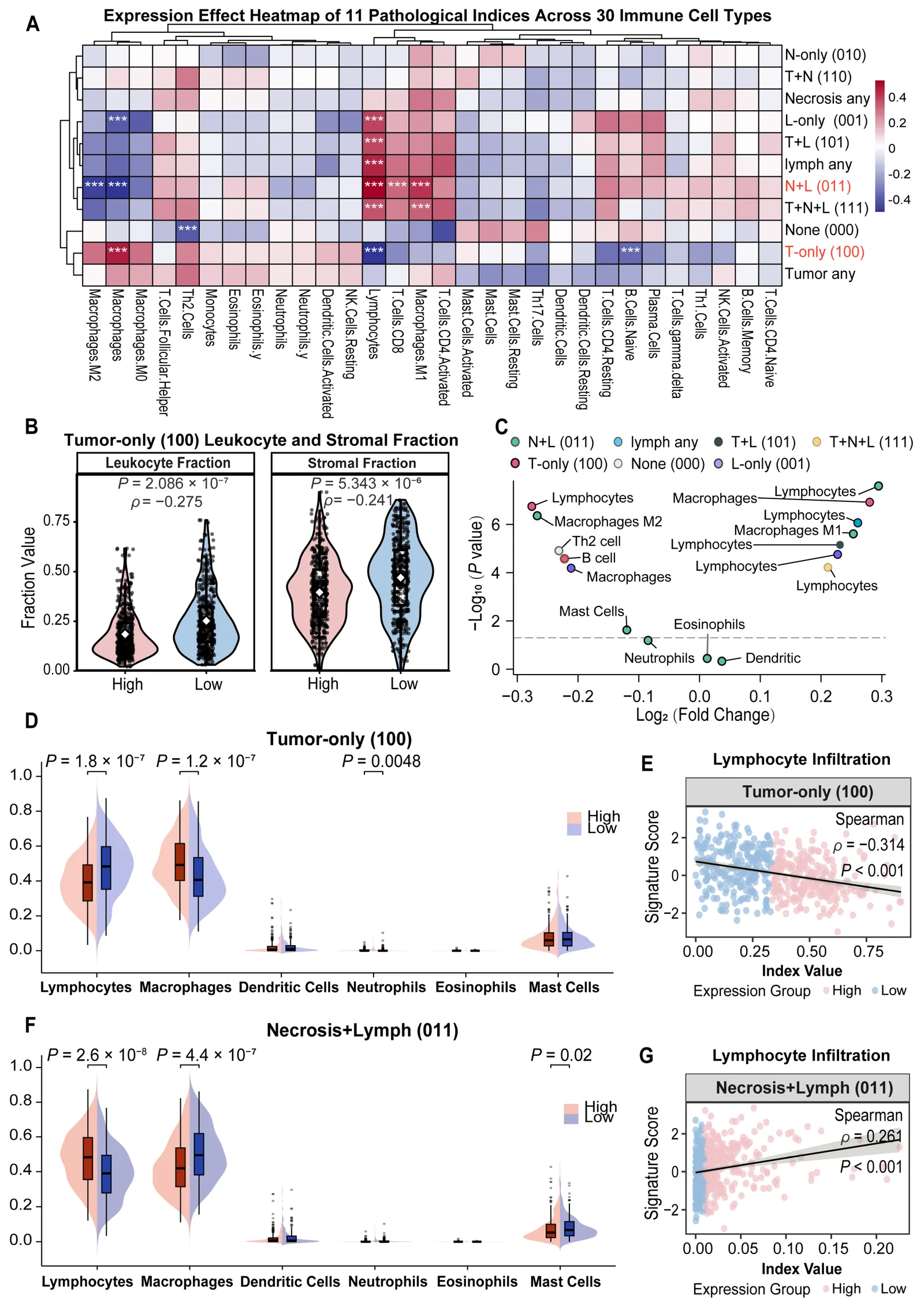
Analysis of immune infiltration characteristics based on 11 pathological features in the TCGA-BRCA cohort. (**A**) Heatmap illustrating the regulatory effects of 11 pathological features across 30 immune cell types. (**B**) Analysis of leukocyte and stromal fractions associated with Pure Tumor-Rich Zone (T-only). (**C**) Volcano plots of immune cell enrichment analysis across the 11 pathological features, depicting differences in immune cell abundance between high- and low-expression groups for each indicator. (**D**) Violin plots showing the distribution of Pure Tumor-Rich Zone (T-only) across key immune cell subsets. (**E**) Spearman correlation analysis between Pure Tumor-Rich Zone (T-only) and the lymphocyte infiltration signature score. (**F**) Violin plots showing the distribution of Lymphocyte-Infiltrated Necrotic Zone (N+L) across key immune cell subsets. (**G**) Spearman correlation analysis between Lymphocyte-Infiltrated Necrotic Zone (N+L) and the lymphocyte infiltration signature score. *** *p* < 0.001.

We further investigated the associations between the pathological features and the tumor immune microenvironment (TIME) in the TCGA-BRCA cohort. Immune cell enrichment analysis across 30 cell types revealed distinct regulatory patterns for different indicators (Figure 7A). Specifically, Pure Tumor-Rich Zone (T-only) exhibited significant negative correlations with both leukocyte fraction (*ρ* = −0.275, *p* = 2.086 × 10^−7^) and stromal fraction (*ρ* = −0.241, *p* = 5.343 × 10^−6^), suggesting that high tumor-region enrichment is inversely associated with immune cell infiltration (Figure 7B).

Volcano plots (Figure 7C) further detailed these immunological characteristics. In the Pure Tumor-Rich Zone (T-only) high-expression group, lymphocyte abundance was significantly decreased (Log_2_FC = −0.276, *p* = 1.81 × 10^−7^), while macrophage abundance was increased (Log_2_FC = 0.280, *p* = 1.22 × 10^−7^). In contrast, the Lymphocyte-Infiltrated Necrotic Zone (N+L) high-expression group showed significantly elevated abundances of lymphocytes (Log_2_FC = 0.295, *p* = 2.60 × 10^−8^) and M1 macrophages (Log_2_FC = 0.251, *p* = 2.17 × 10^−6^), whereas M2 macrophage abundance was markedly reduced (Log_2_FC = −0.276, *p* = 1.86 × 10^−7^). These findings suggest that Lymphocyte-Infiltrated Necrotic Zone (N+L) enrichment is associated with a pro-inflammatory microenvironment. Other indicators, such as Global Lymphoid Compartment (L-any) and Tumor–Lymphocyte Coexistence (T+L), also showed increased lymphocyte abundance but with smaller effect sizes than Lymphocyte-Infiltrated Necrotic Zone (N+L).

Subgroup analysis confirmed that the Pure Tumor-Rich Zone (T-only) high-expression group was characterized by low lymphocyte expression (*p =* 1.8 × 10^−7^), high macrophage expression (*p =* 1.2 × 10^−7^), and low neutrophil expression (*p* = 0.0048) (Figure 7D). Conversely, Lymphocyte-Infiltrated Necrotic Zone (N+L) exhibited a significant immune profile characterized by high lymphocyte (*p* = 2.6 × 10^−8^), low macrophage (*p* = 4.4 × 10^−7^) infiltration, and low mast cell expression (*p* = 0.02) (Figure 7F). Correlation analysis verified these trends: Pure Tumor-Rich Zone (T-only) was negatively correlated with the lymphocyte infiltration score (*ρ* = −0.314, *p* < 0.001), while Lymphocyte-Infiltrated Necrotic Zone (N+L) showed a positive correlation (*ρ* = 0.261, *p* < 0.001) (Figure 7E,G).

Collectively, these results indicate that our pathological features effectively reflect the heterogeneity of the breast cancer immune microenvironment, providing evidence for their potential role in assessing immunotherapeutic response.

### 3.8. Exploratory Prognostic Association and Molecular Mechanism Analysis of Key Pathological Features

**Figure 8:**
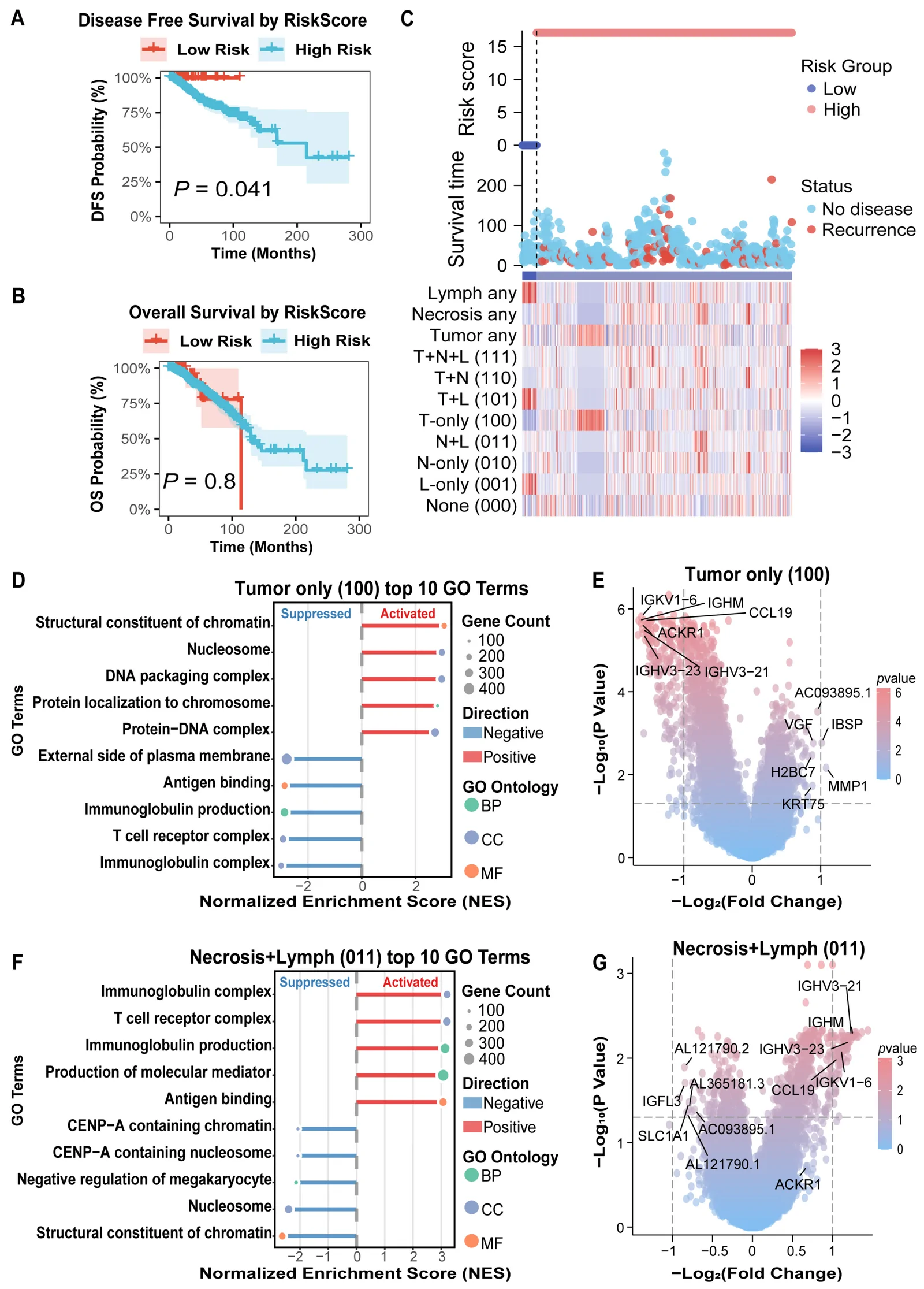
Prognostic risk stratification and molecular mechanism analysis of key pathological features in the TCGA-BRCA cohort. (**A**) DFS curves based on RiskScore in the TCGA-BRCA cohort. (**B**) OS curves based on RiskScore in the TCGA-BRCA cohort. (**C**) DFS prognostic risk score and feature heatmap constructed based on pathological features. (**D**) GO terms significantly enriched in patients with high Pure Tumor-Rich Zone (T-only) expression. (**E**) Volcano plot of differentially expressed genes comparing high versus low Pure Tumor-Rich Zone (T-only) expression groups. (**F**) GO terms significantly enriched in patients with high Lymphocyte-Infiltrated Necrotic Zone (N+L) expression. (**G**) Volcano plot of differentially expressed genes comparing high versus low Lymphocyte-Infiltrated Necrotic Zone (N+L) expression groups.

A prognostic risk score was constructed based on the pathological features to stratify patients in the TCGA-BRCA cohort (Figure 8A–C). The results showed a significant difference in disease-free survival (DFS) between the high-risk and low-risk groups (*p* = 0.041), whereas no statistically significant difference was observed in overall survival (OS) (*p* = 0.8). This suggests that pathological features primarily serve as potential indicators for estimating DFS. The distribution of risk scores, recurrence status, and the expression heatmap further illustrated the correlation between high-risk scores and recurrence events, as well as the differential expression patterns of pathological features across risk groups (Figure 8C).

To elucidate the potential molecular mechanisms, we prioritized Pure Tumor-Rich Zone (T-only) and Lymphocyte-Infiltrated Necrotic Zone (N+L) for differential expression and gene enrichment analyses (Figure 8D–G). These two features were selected because they represent direct pathological counterparts in terms of composition, with one being purely tumor and the other consisting of necrosis with lymphocyte infiltration, allowing us to explore the opposing tumor microenvironment effects associated with these contrasting histological patterns.

GO enrichment analysis revealed that the Pure Tumor-Rich Zone (T-only) high-expression group was significantly enriched in pathways related to chromatin structure remodeling (Figure 8D), including structural constituent of chromatin, nucleosome, DNA packaging complex, and protein-DNA complex. Volcano plots (Figure 8E) further identified multiple differentially expressed genes: immunoglobulin family genes (e.g., *IGHM*, *IGKV1-6*, *IGHV3-23*) and *CCL19* were significantly downregulated, whereas tumor proliferation-related genes (e.g., *VGF*, *IBSP*, *MMP1*, *H2BC7*, and *KRT75*) were significantly upregulated in the Pure Tumor-Rich Zone (T-only) high-expression group.

In contrast, the Lymphocyte-Infiltrated Necrotic Zone (N+L) high-expression group was significantly enriched in immune activation-related pathways (Figure 8F), such as the immunoglobulin complex, T cell receptor complex, and antigen binding. Differential gene analysis (Figure 8G) showed that immune-related genes (e.g., *IGHM*, *IGKV1-6*, *IGHV3-23*) and *CCL19* were significantly upregulated, while tumorigenesis-related genes (e.g., *SLC1A1*, *IGFL3*, *AL121790.1*, and *AC093895.1*) were significantly downregulated. This molecular pattern is notably opposite to that of Pure Tumor-Rich Zone (T-only).

Overall, these findings indicate that Pure Tumor-Rich Zone (T-only) and Lymphocyte-Infiltrated Necrotic Zone (N+L) modulate the immune microenvironment and prognosis through distinct molecular pathways, providing key clues for the discriminative value of spatial pathological features in breast cancer.

## 4. Discussion

This study explores the practical utility of spatial PIFs in assessing RCB following NAT for breast cancer. While deep learning models continue to improve in discriminative accuracy, their internal mechanisms often remain opaque to clinicians [40,41,42]. Common deep learning approaches in digital pathology include slide-level weakly supervised models (e.g., ABMIL, CLAM) and patch-level foundation models (e.g., UNI, Phikon, Virchow) [16,38,43,44,45]. These methods are predominantly data-driven with limited interpretability, making the rationale behind their assessments difficult for pathologists to directly interpret and validate [35,43,45,46]. In contrast, our study leverages deep learning backbones (VGG-16, ResNet-50, and ViT-Base) to extract patch-level features, integrated with CLAM for tissue component classification [43,47,48]. By defining spatial PIFs based on these components and combining them with SHAP analysis, we effectively translated complex high-dimensional data into observable morphological patterns involving tumor, necrosis, and lymphocytes. This approach not only provides accurate RCB assessments but also offers a histological rationale for the judgment by revealing associations with therapy response, immune infiltration, and molecular profiles [13,26,37]. Prior to spatial feature construction, we compared VGG-16, ResNet-50 and ViT-Base for patch-level histological classification [47,48]. VGG-16 outperformed ResNet-50 and ViT-Base in patch classification, likely because its shallower architecture better fitted the limited training data, its convolutional inductive biases suit histopathological pattern recognition, and its low-level ImageNet features transfer more effectively than deeper semantic representations [47,48,49]. In contrast, ResNet-50′s large capacity risks overfitting, while ViT-Base relied on global attention and underperformed with limited annotations due to insufficient spatial priors [44,48,49]. Accordingly, we adopted VGG-16 as the patch-level feature extraction backbone for subsequent spatial PIF construction.

The clinical value of the RCB system depends on its ability to accurately reflect the extent of residual disease [9,33]. Current research frequently focuses on estimating pathologic complete response (pCR), which serves only as a binary endpoint. However, patients who do not achieve pCR exhibit significant heterogeneity, with RCB grades I, II, and III carrying markedly different risks of recurrence [9,10,11,50]. Most existing models utilize only binary pCR status as the study endpoint, failing to capture the nuanced differences across the continuous spectrum of residual burden [14,22,51]. Furthermore, traditional artificial intelligence models often face limitations such as single-feature dimensionality and a lack of clinical interpretability [13,52]. In contrast, our study addresses the four-category RCB system by implementing three progressive classification schemes, a design aimed at better capturing the inherent clinical heterogeneity among non-pCR patients [51]. As established by Symmans et al. [33], incremental increases in the RCB score are closely associated with recurrence risk; specifically, the accurate differentiation between low and intermediate-to-high residual burdens is pivotal for clinical decisions regarding adjuvant therapy intensification [14,22]. Our results demonstrate that models based on XGBoost achieve stable performance in distinguishing these RCB grades. We hypothesize that the spatial arrangement of tumor, necrosis, and lymphocyte regions may provide additional discriminative information beyond what is captured by cellular density alone [21,37].

While traditional RCB scoring remains the benchmark for quantifying residual disease, its methodology primarily relies on the linear assessment of individual pathological components, which fails to account for the complex spatial interdependencies within the TME [15,53]. Our study addresses this limitation by constructing a panel of spatial PIFs that integrate the co-occurrence patterns of tumor cells, necrosis, and lymphocytes. A key finding of this study is the divergent roles of two core pathological signatures: Pure Tumor-Rich Zone (T-only) and Lymphocyte-Infiltrated Necrotic Zone (N+L). SHAP analysis identified Pure Tumor-Rich Zone (T-only) as a primary driver of high residual tumor burden. Histologically, this corresponds to a classic “immune desert” phenotype [53], characterized by a lack of immune response surrounding tumor nests, suggesting a failure of the therapy to elicit an effective anti-tumor immune response [54].

In contrast, the enrichment of Lymphocyte-Infiltrated Necrotic Zone (N+L) was significantly associated with pCR and lower residual burden, suggesting that lymphocyte infiltration around necrotic regions may reflect active tumor regression, although this interpretation should be weighed against the potential confounding of hyalinised fibrosis in post-treatment tissues [55,56]. Current tumor-infiltrating lymphocytes (TILs) evaluation systems generally neglect the spatial architecture and distribution patterns of immune cells within the TME [57,58]. Traditional TILs scoring often lacks spatial context, making it difficult to determine whether lymphocytes have effectively reached the “site” of tumor cell death [57,58,59]. This spatial PIF captures the spatial accompaniment between lymphocytes and necrotic debris, offering a quantitative dimension that traditional cell counting lacks [60]. This spatial relationship may reflect specific histological patterns of tumor-immune interaction during neoadjuvant therapy, which potentially contributes to the model’s discriminative accuracy for RCB stratification [11,61]. These morphology-based spatial pathological features not only identify localized therapy-induced active microenvironments but also provide a more biologically interpretable metric for assessing therapeutic efficacy by capturing the intricate interactions between tumor, necrotic, and lymphocyte regions [62]. Previous studies have confirmed the prognostic value of spatial TIL distribution and tumor boundary metrics [37,63].

Building upon these paradigms, our study further extends spatial analysis to the RCB scoring system, demonstrating that the physical co-occurrence patterns of multiple tissue components offer more profound insights into therapy-induced regression than those provided by isolated cellular densities.

Validation against immune infiltration and molecular pathway data from the TCGA-BRCA cohort indicated that these morphological differences reflect the functional status of the microenvironment. The association of Lymphocyte-Infiltrated Necrotic Zone (N+L) with M1-type macrophage enrichment and immune-activation pathways supports its role as a pro-inflammatory marker. Conversely, the Pure Tumor-Rich Zone (T-only) signature was linked to chromatin remodeling and an immunosuppressive environment characterized by macrophage enrichment and lymphocyte depletion. These findings suggest that spatial analysis of routine H&E sections may allow for a preliminary inference of the tumor immune status without the need for expensive genomic sequencing. Distinct from frameworks such as MISO that rely on costly spatial omics data for multiscale integration, our results suggest that routine morphological features may harbor valuable molecular regulatory information [62]. Specifically, the spatial patterns identified by our model exhibit associations with key biological processes, such as M1/M2 macrophage polarization within the microenvironment, which have been reported to influence chemosensitivity and immune activation [64,65]. This observed correlation between morphology and molecular status hints at the potential of spatial interaction analysis on ubiquitous H&E sections to provide a cost-effective and supplementary perspective for assessing the functional immune landscape [62,66].

Integrating multi-component spatial features with XAI, our framework enabled clinically aligned interpretability. Tissue segmentation provided a spatial foundation for feature localization, MIL eliminated patch-level annotations, and feature engineering translated pathological knowledge into quantitative metrics, with SHAP and Grad-CAM offering complementary explanations [35,48,67,68]. Despite limitations in segmentation accuracy, feature-space constraints, and computational cost, these were partially offset by cross-module synergy [43,48,67]. Collectively, spatial PIFs hold promise as histopathological biomarkers for objective RCB stratification, aiding in identifying patients who may benefit from intensified therapy [19,28,29,30].

Despite these findings, several limitations remain. First, the retrospective nature of the study introduces potential selection bias, and the model’s generalizability requires further validation across multi-center data. Second, the observed correlations between morphology and molecules do not imply causality. Future research using spatial transcriptomics is needed to verify gene expression differences at a more microscopic level. Additionally, the molecular subtype distribution in our internal cohort was imbalanced, with the HER2-any staining subgroup accounting for 169 of 182 cases, which precluded reliable subtype-stratified analyses of feature-to-RCB associations. Third, several models achieved near-perfect discriminative performance for Task 3, which may partly reflect the limited sample size as well as marked morphological distinction between the RCB-3 group (extensive residual tumor) and the RCB-0+1+2 group (tumor regression). Future work will expand the sample size to validate the robustness of these findings. Fourth, we acknowledge that hyalinised fibrosis in post-treatment tissues may have been morphologically misclassified as necrosis, which should be considered when interpreting features involving the necrosis compartment.

In summary, the extraction and quantitative analysis of spatial PIFs provide detailed insights into therapy response in breast cancer. These findings offer a new perspective on how morphological patterns reflect immune status and prognosis, contributing to the development of personalized therapeutic strategies.

## 5. Conclusions

In summary, this study established spatial pathological interpretable features for quantitative RCB stratification in breast cancer following neoadjuvant therapy. SHAP analysis identified Necrotic Tumor Mixed Cluster (T+N) and Pure Necrotic-Rich Zone (N-only) as the predominant indicators across the three RCB tasks. In exploratory TCGA association analyses, tumor-rich zones and lymphocyte-infiltrated necrotic zones correlated with opposing immune phenotypes. Although prospective validation is warranted, our findings suggest that quantifying the spatial pathological features of residual disease may complement routine pathological evaluation and inform personalized clinical decision-making.

## Figures and Tables

**Table 1:** Baseline characteristics of the training, test, and external validation sets.

Characteristics	Training Set	Test Set	External Validation Set
**Number of patients**	147	35	165
**Median age, years**	49	54	50
**Tumor laterality**			
Left breast	70 (47.62%)	17 (48.57%)	79 (47.88%)
Right breast	74 (50.34%)	18 (51.43%)	83 (50.30%)
Bilateral	3 (2.04%)	0 (0.00%)	3 (1.82%)
**RCB**			
0	39 (26.53%)	9 (25.71%)	40 (24.24%)
I	35 (23.81%)	9 (25.71%)	43 (26.06%)
II	39 (26.53%)	5 (14.29%)	41 (24.85%)
III	34 (23.13%)	12 (34.29%)	41 (24.85%)
**Ki-67**			
≤20%	30 (20.41%)	6 (17.14%)	32 (19.39%)
>20%	117 (79.59%)	29 (82.86%)	133 (80.61%)
**ER**			
Absent	63 (42.86%)	17 (48.57%)	74 (44.85%)
Present	84 (57.14%)	18 (51.43%)	91 (55.15%)
**PR**			
Absent	83 (56.46%)	23 (65.71%)	98 (59.39%)
Present	64 (43.54%)	12 (34.29%)	67 (40.61%)
**AR**			
Absent	23 (15.65%)	6 (17.14%)	29 (17.58%)
Present	124 (84.35%)	29 (82.86%)	136 (82.42%)
**HER2**			
IHC 0	11 (7.48%)	2 (5.71%)	13 (7.88%)
Any staining	136 (92.52%)	33 (94.29%)	152 (92.12%)

**Table 2:** Definitions of 11 spatial pathological features and tissue regions.

Features Code	Standard Morphological Name (Abbreviation)	Tissue Regions and Morphological Definition
Comb 000	None	Normal tissue region without tumor, necrosis, and lymphocyte infiltration.
Comb 001	Pure Lymphoid-Rich Zone (L-only)	Pure immune region with only lymphocyte infiltration, absence of tumor and necrosis components.
Comb 010	Pure Necrotic-Rich Zone (N-only)	Pure necrotic tissue region without tumor and immune infiltration.
Comb 100	Pure Tumor-Rich Zone (T-only)	Pure tumor parenchyma region without necrosis or lymphocyte infiltration.
Comb 011	Lymphocyte-Infiltrated Necrotic Zone (N+L)	Region with co-localization of necrotic debris and infiltrating lymphocytes.
Comb 101	Tumor–Lymphocyte Coexistence (T+L)	Region with coexistence of tumor and lymphocyte.
Comb 110	Necrotic Tumor Mixed Cluster (T+N)	Region with coexistence of tumor and necrosis.
Comb 111	Triple-Coexistence Zone (T+N+L)	Region with coexistence of tumor, necrosis, and lymphocyte.
Total tumor	Global Tumor Compartment (T-any)	Sum of all regions containing tumor.
Total necrosis	Global Necrotic Compartment (N-any)	Sum of all regions containing necrosis.
Total lymphocyte	Global Lymphoid Compartment (L-any)	Sum of all regions containing lymphocyte.

## Data Availability

The data that support the findings of this study are derived from two sources. The publicly available TCGA-BRCA cohort data can be accessed at https://portal.gdc.cancer.gov/projects/TCGA-BRCA (accessed on 1 January 2025). The data from the internal clinical cohorts are not publicly available due to patient privacy and institutional review board restrictions; however, de-identified data and the analysis code are available from the corresponding author upon reasonable request.

## Supplementary Materials

The following supporting information can be downloaded at https://www.mdpi.com/article/10.3390/diagnostics16172798/s1, Figure S1. Detailed performance metrics of three deep learning models for core pathological region identification; Figure S2. LASSO variable selection results for three binary RCB stratification tasks based on the eight combinatorial features, with comb 000 excluded from the feature set to avoid exact collinearity; Figure S3. Radar charts and comprehensive performance comparison of six ML models (Bayesian, XGBoost, KNN, NN, RF, and logistic regression) in the external validation set for three binary RCB stratification tasks; Figure S4. Actual versus predicted RCB score plots of six machine learning models in the training, test, and external validation sets; Figure S5. SHAP dependence plots of core features in Task 1 (RCB 0 vs RCB 1+2+3); Figure S6. SHAP dependence plots of core features in Task 2 (RCB 0+1 vs RCB 2+3); Figure S7. SHAP dependence plots of core features in Task 3 (RCB 0+1+2 vs RCB 3); Figure S8. (A) Distribution of the Pure Necrotic-Rich Zone (N-only) feature across RCB subgroups in the internal cohort (*N* = 182). (B) Heatmap of Spearman correlation coefficients between 11 spatial pathological features and four molecular subtypes (HER2-any staining, Luminal A, Luminal B, and TNBC) in the internal cohort (*N* = 182). Each cell represents the correlation coefficient (ρ), with red indicating positive correlation and blue indicating negative correlation. Asterisks denote statistical significance. (C) Boxplots comparing the distribution of 11 pathological features between HER2-any staining and HER2-IHC 0 subgroups in the internal cohort (*N* = 182). p values from Mann-Whitney U tests are shown; no significant differences were observed (all *p* > 0.05). (D) Heatmap of Z-score normalized median expression of 11 pathological features across RCB grades (0, 1, 2, 3) within the HER2-any staining subgroup (*n* = 169) of the internal cohort. Asterisks indicate significance from Kruskal-Wallis tests (*** *p* < 0.001, ** *p* < 0.01, * *p* < 0.05). Figure S9. Calibration curves of six machine learning models in training, internal test and external validation datasets; Table S1. AUC values with 95% CIs for the six machine learning models in the three tasks; Table S2. Spearman rank correlation between 11 spatial pathological features and model-estimated RCB-like scores in the TCGA-BRCA cohort (exploratory analysis).
