# Supplementary material for: Explainable AI-Derived Spatial Pathological Features of Tumor, Necrosis, and Lymphocytes Identify Key Histological Signatures for Residual Cancer Burden Assessment in Breast Cancer
Source: Diagnostics (Basel). 2026 Aug 31;16(17):2798. doi: 10.3390/diagnostics16172798 (PMC13564659; doi:10.3390/diagnostics16172798)
Supplement: Supplementary file 1 [file diagnostics-16-02798-s001.zip › diagnostics-4446529-supplementary.pdf]

Supplementary Information

Supplementary Figures S1-S9

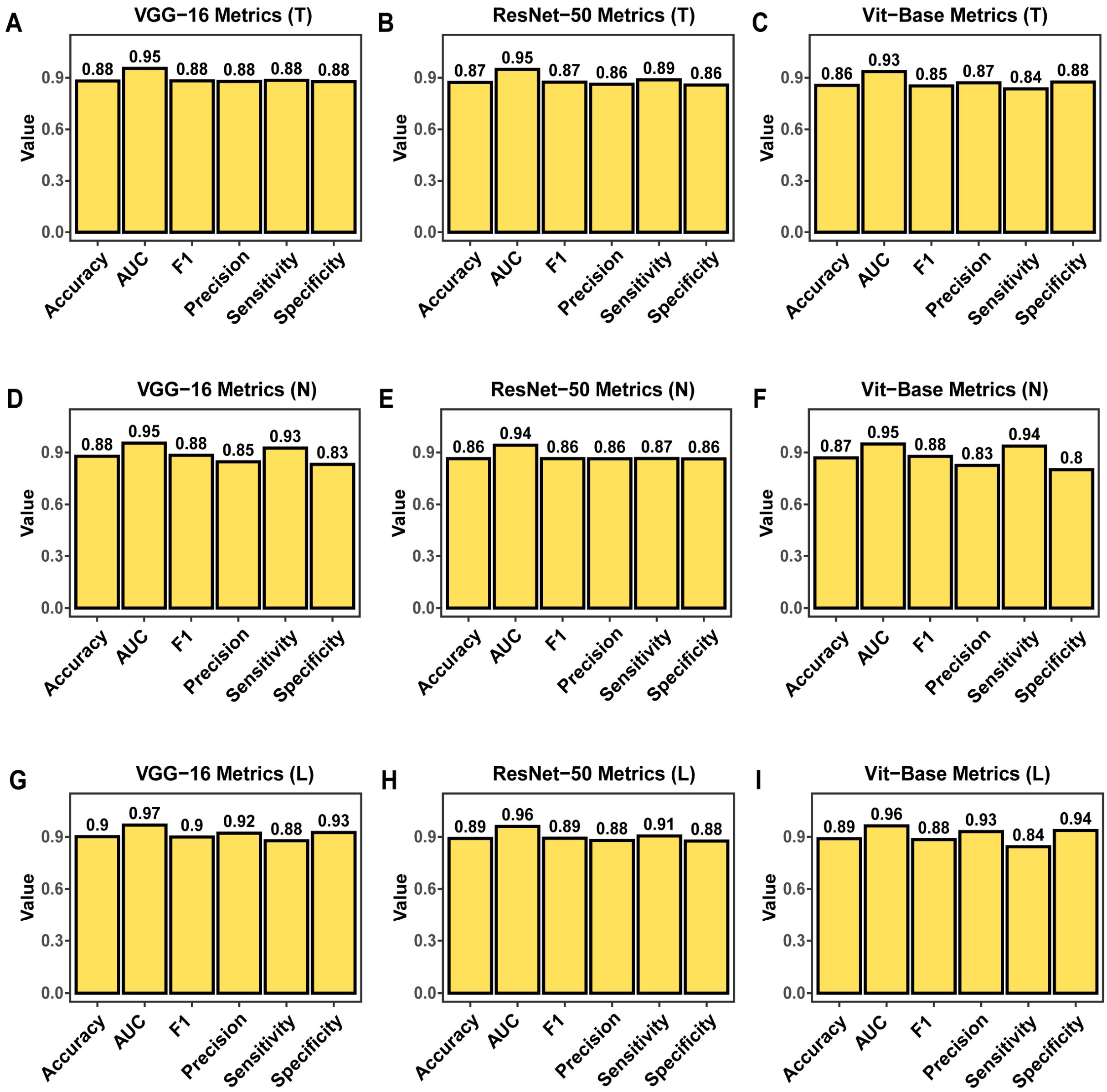

Figure S1. Detailed performance metrics of three deep learning models for core pathological region identification. **A.** Bar chart of VGG-16 performance metrics for tumor region identification. **B.** Bar chart of ResNet-50 performance metrics for tumor region identification. **C.** Bar chart of ViT-Base performance metrics for tumor region identification. **D.** Bar chart of VGG-16 performance metrics for necrosis region identification. **E.** Bar chart of ResNet-50 performance metrics for necrosis region identification. F. Bar chart of ViT-Base performance metrics for necrosis region identification. **G.** Bar chart of VGG-16 performance metrics for lymphocyte region identification. **H.** Bar chart of ResNet-50 performance metrics for lymphocyte region identification. I. Bar chart of ViT-Base performance metrics for lymphocyte region identification.

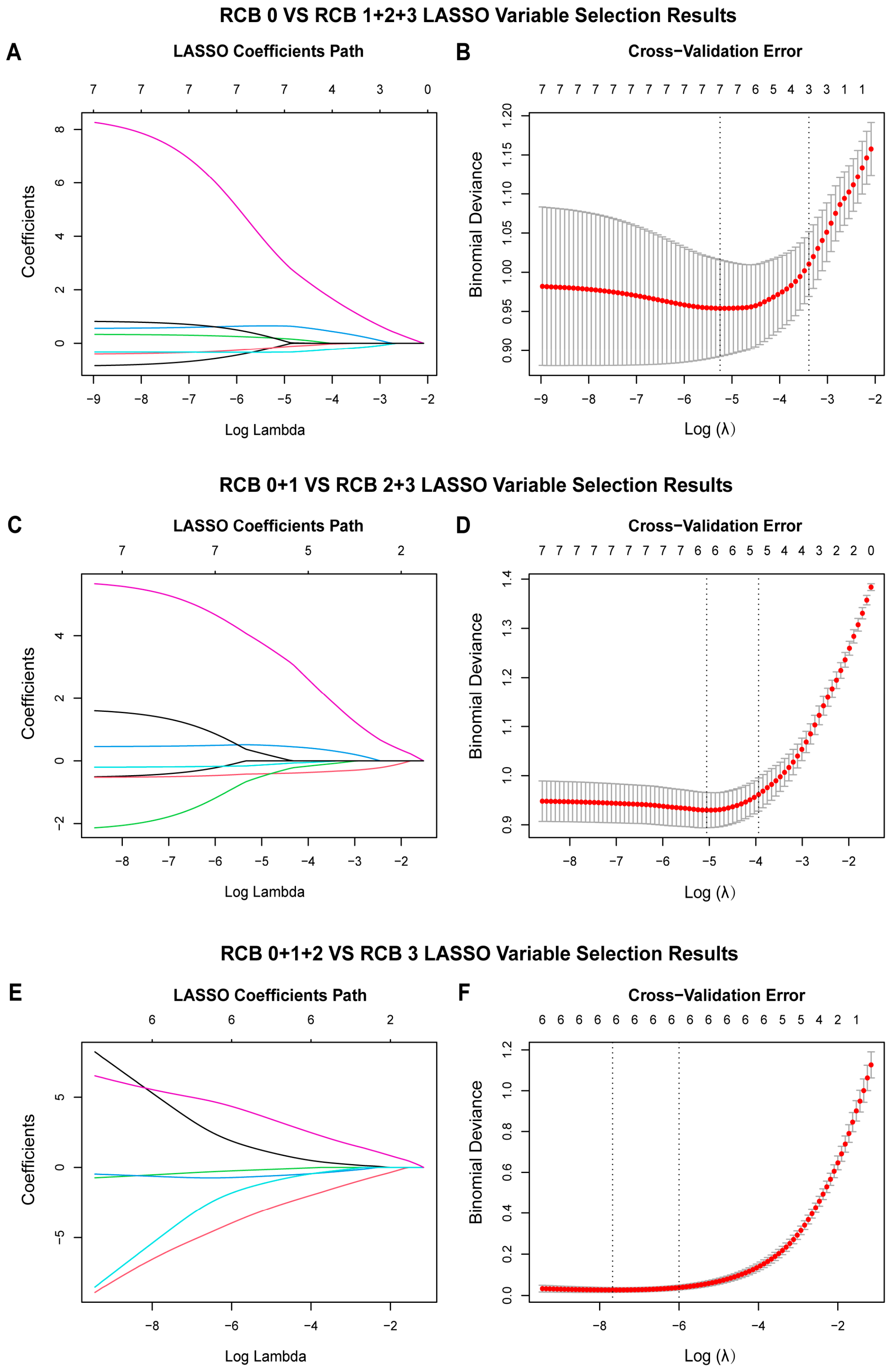
RCB 0 VS RCB 1+2+3 LASSO Variable Selection Results
A
LASSO Coefficients Path
7 7 7 7 7 4 3 0
Coefficients
Log Lambda
B
Cross−Validation Error
7 7 7 7 7 7 7 7 7 7 7 7 6 5 4 3 3 1 1
Binomial Deviance
Log (λ)
RCB 0+1 VS RCB 2+3 LASSO Variable Selection Results
C
LASSO Coefficients Path
7 7 5 2
Coefficients
Log Lambda
D
Cross−Validation Error
7 7 7 7 7 7 7 7 7 6 6 6 5 5 4 4 3 2 2 0
Binomial Deviance
Log (λ)
RCB 0+1+2 VS RCB 3 LASSO Variable Selection Results
E
LASSO Coefficients Path
6 6 6 2
Coefficients
Log Lambda
F
Cross−Validation Error
6 6 6 6 6 6 6 6 6 6 6 6 6 5 5 4 2 1
Binomial Deviance
Log (λ)

Figure S2. LASSO variable selection results for three binary RCB stratification tasks based on the eight combinatorial features, with comb 000 excluded from the feature set to avoid exact collinearity. **A.** LASSO coefficient path plot for Task 1 (RCB 0 vs RCB 1+2+3). **B.** Cross-validation binomial deviance curve for Task 1 (RCB 0 vs RCB 1+2+3). **C.** LASSO coefficient path plot for Task 2 (RCB 0+1 vs RCB 2+3). **D.** Cross-validation binomial deviance curve for Task 2 (RCB 0+1 vs RCB 2+3). **E.** LASSO coefficient path plot for Task 3 (RCB 0+1+2 vs RCB 3). **F.** Cross-validation binomial deviance curve for Task 3 (RCB 0+1+2 vs RCB 3).

A

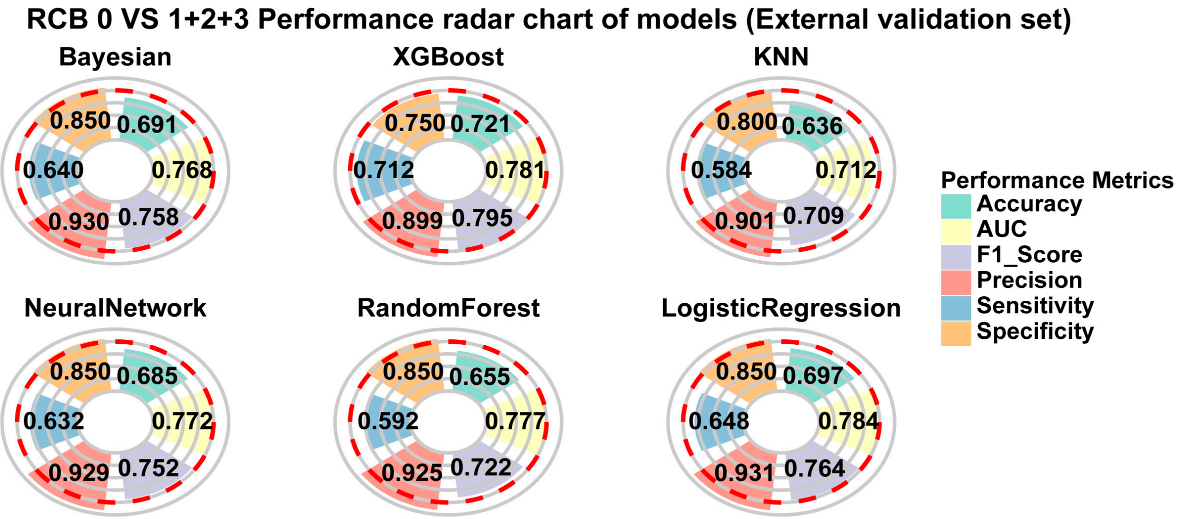
RCB 0 VS 1+2+3 Performance radar chart of models (External validation set)
Bayesian
0.850 0.691 0.640 0.768 0.930 0.758
XGBoost
0.750 0.721 0.712 0.781 0.899 0.795
KNN
0.800 0.636 0.584 0.712 0.901 0.709
NeuralNetwork
0.850 0.685 0.632 0.772 0.929 0.752
RandomForest
0.850 0.655 0.592 0.777 0.925 0.722
LogisticRegression
0.850 0.697 0.648 0.784 0.931 0.764
Performance Metrics
Accuracy
AUC
F1_Score
Precision
Sensitivity
Specificity

B

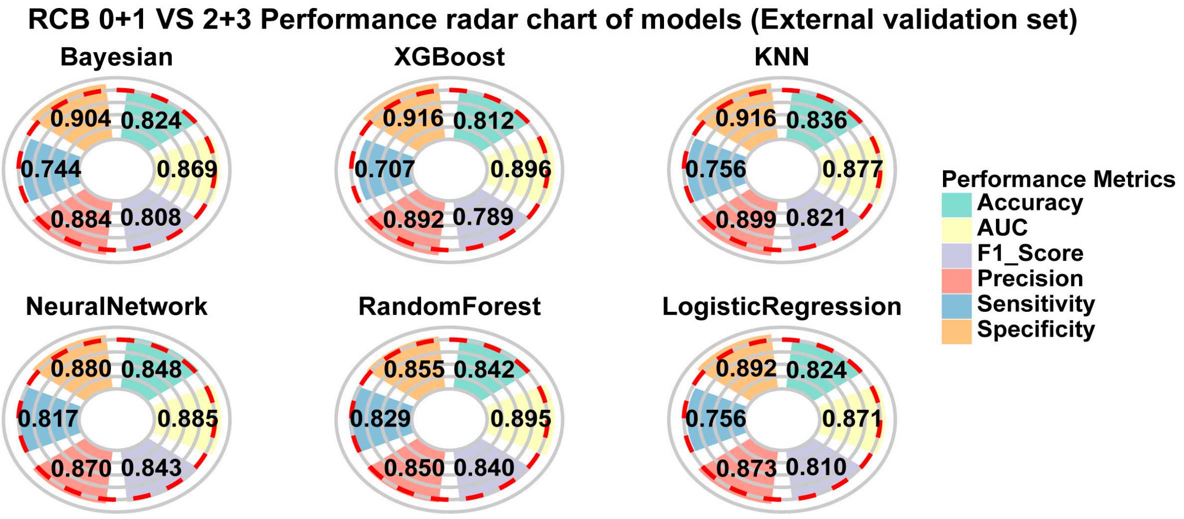
RCB 0+1 VS 2+3 Performance radar chart of models (External validation set)
Bayesian
0.904 0.824 0.744 0.869 0.884 0.808
XGBoost
0.916 0.812 0.707 0.896 0.892 0.789
KNN
0.916 0.836 0.756 0.877 0.899 0.821
NeuralNetwork
0.880 0.848 0.817 0.885 0.870 0.843
RandomForest
0.855 0.842 0.829 0.895 0.850 0.840
LogisticRegression
0.892 0.824 0.756 0.871 0.873 0.810
Performance Metrics
Accuracy
AUC
F1_Score
Precision
Sensitivity
Specificity

C

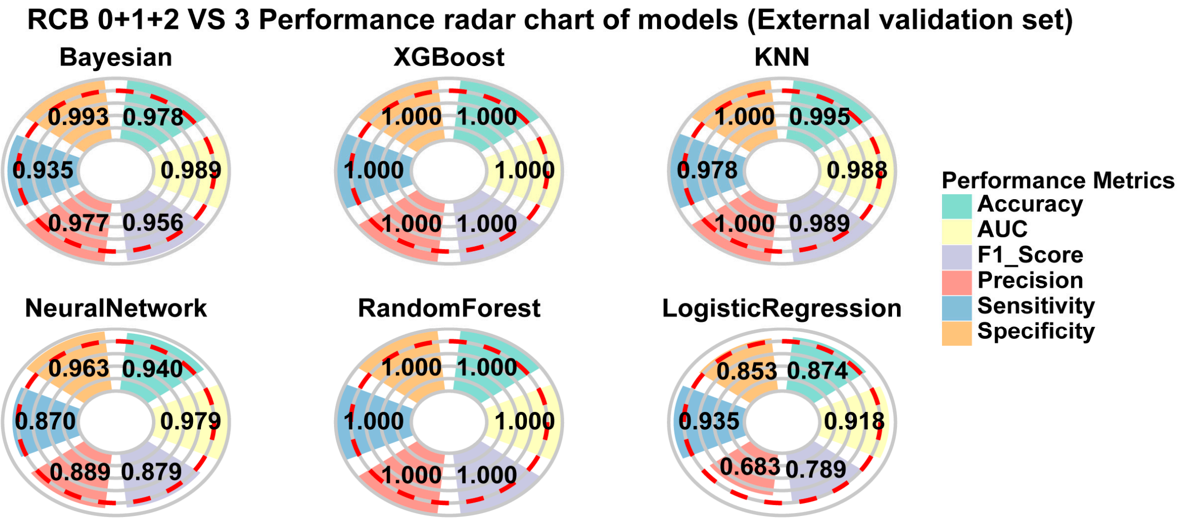
RCB 0+1+2 VS 3 Performance radar chart of models (External validation set)
Bayesian
0.993 0.978 0.935 0.989 0.977 0.956
XGBoost
1.000 1.000 1.000 1.000 1.000 1.000
KNN
1.000 0.995 0.978 0.988 1.000 0.989
NeuralNetwork
0.963 0.940 0.870 0.979 0.889 0.879
RandomForest
1.000 1.000 1.000 1.000 1.000 1.000
LogisticRegression
0.853 0.874 0.935 0.918 0.683 0.789
Performance Metrics
Accuracy
AUC
F1_Score
Precision
Sensitivity
Specificity

Figure S3**.** Radar charts and comprehensive performance comparison of six ML models (Bayesian, XGBoost, KNN, NN, RF, and logistic regression) in the external validation set for three binary RCB stratification tasks. **A.** Radar chart showing the comprehensive performance of six ML models in Task 1 (RCB 0 vs RCB 1+2+3), including key metrics such as accuracy, AUC, F1 score, precision, sensitivity, and specificity. **B.** Radar chart showing the comprehensive performance of six ML models in Task 2 (RCB 0+1 vs RCB 2+3), including key metrics such as accuracy, AUC, F1 score, precision, sensitivity, and specificity. **C.** Radar chart showing the comprehensive performance of six ML models in Task 3 (RCB 0+1+2 vs RCB 3), including key metrics such as accuracy, AUC, F1 score, precision, sensitivity, and specificity.

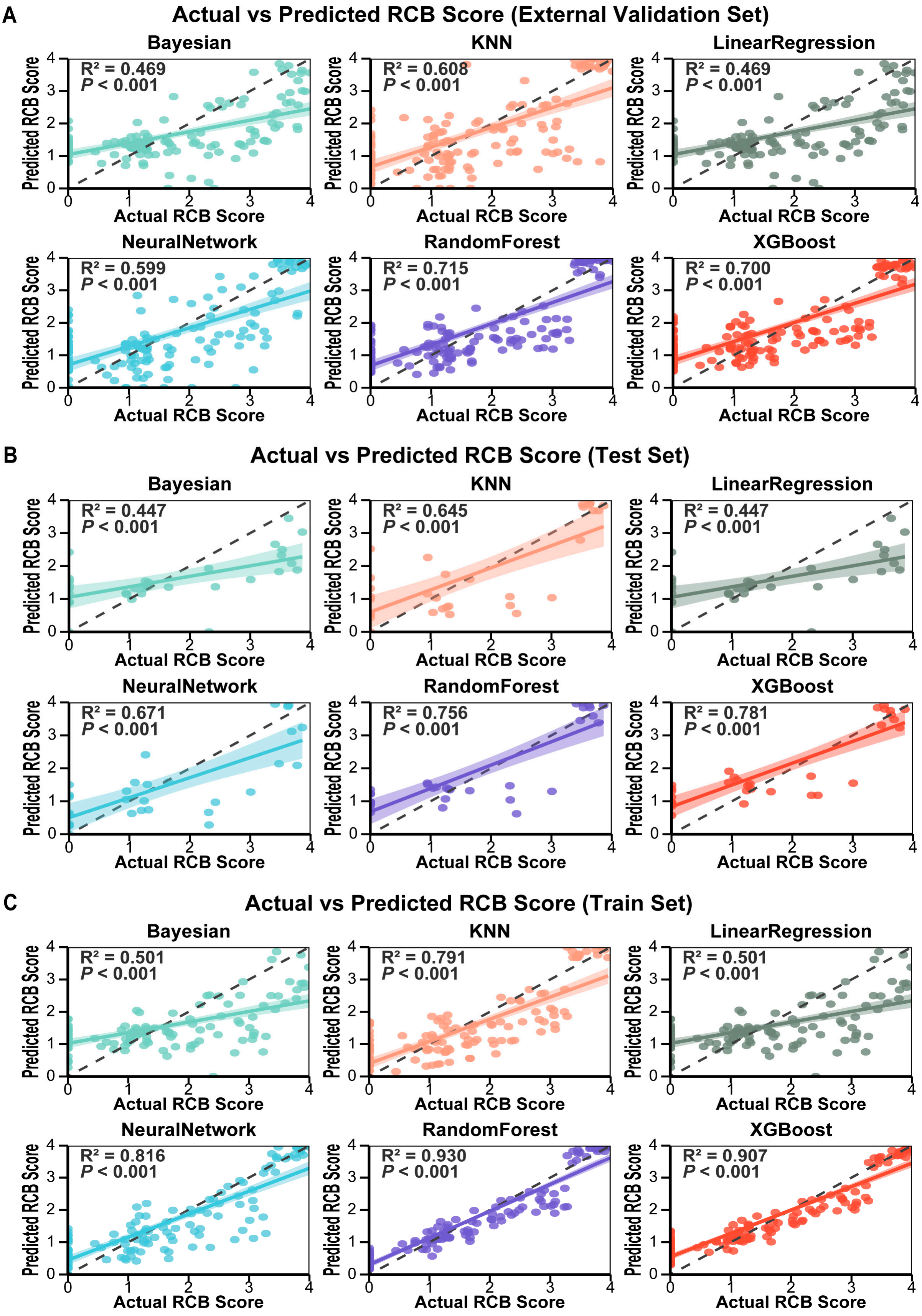
A
Actual vs Predicted RCB Score (External Validation Set)
Bayesian
R² = 0.469
P < 0.001
KNN
R² = 0.608
P < 0.001
LinearRegression
R² = 0.469
P < 0.001
NeuralNetwork
R² = 0.599
P < 0.001
RandomForest
R² = 0.715
P < 0.001
XGBoost
R² = 0.700
P < 0.001
B
Actual vs Predicted RCB Score (Test Set)
Bayesian
R² = 0.447
P < 0.001
KNN
R² = 0.645
P < 0.001
LinearRegression
R² = 0.447
P < 0.001
NeuralNetwork
R² = 0.671
P < 0.001
RandomForest
R² = 0.756
P < 0.001
XGBoost
R² = 0.781
P < 0.001
C
Actual vs Predicted RCB Score (Train Set)
Bayesian
R² = 0.501
P < 0.001
KNN
R² = 0.791
P < 0.001
LinearRegression
R² = 0.501
P < 0.001
NeuralNetwork
R² = 0.816
P < 0.001
RandomForest
R² = 0.930
P < 0.001
XGBoost
R² = 0.907
P < 0.001
Predicted RCB Score
Actual RCB Score
0
1
2
3
4

Figure S4. Actual versus predicted RCB score plots of six machine learning models in the training, test, and external validation sets. Scatter plots with regression lines illustrate the correlation between actual and predicted residual cancer burden (RCB) scores for six ML models (Bayesian, KNN, linear regression, NN, RF, and XGBoost) across three datasets. **A.** Actual versus predicted RCB score in the external validation set: scatter plots of predicted versus actual RCB scores for six ML models in an independent external validation cohort. **B.** Actual versus predicted RCB score in the test set: scatter plots of predicted versus actual RCB scores for the same six ML models in the held-out test set. **C.** Actual versus predicted RCB score in the training set: scatter plots of predicted versus actual RCB scores for the six ML models in the training set. Each plot displays the coefficient of determination (R^2) and *P*-value.

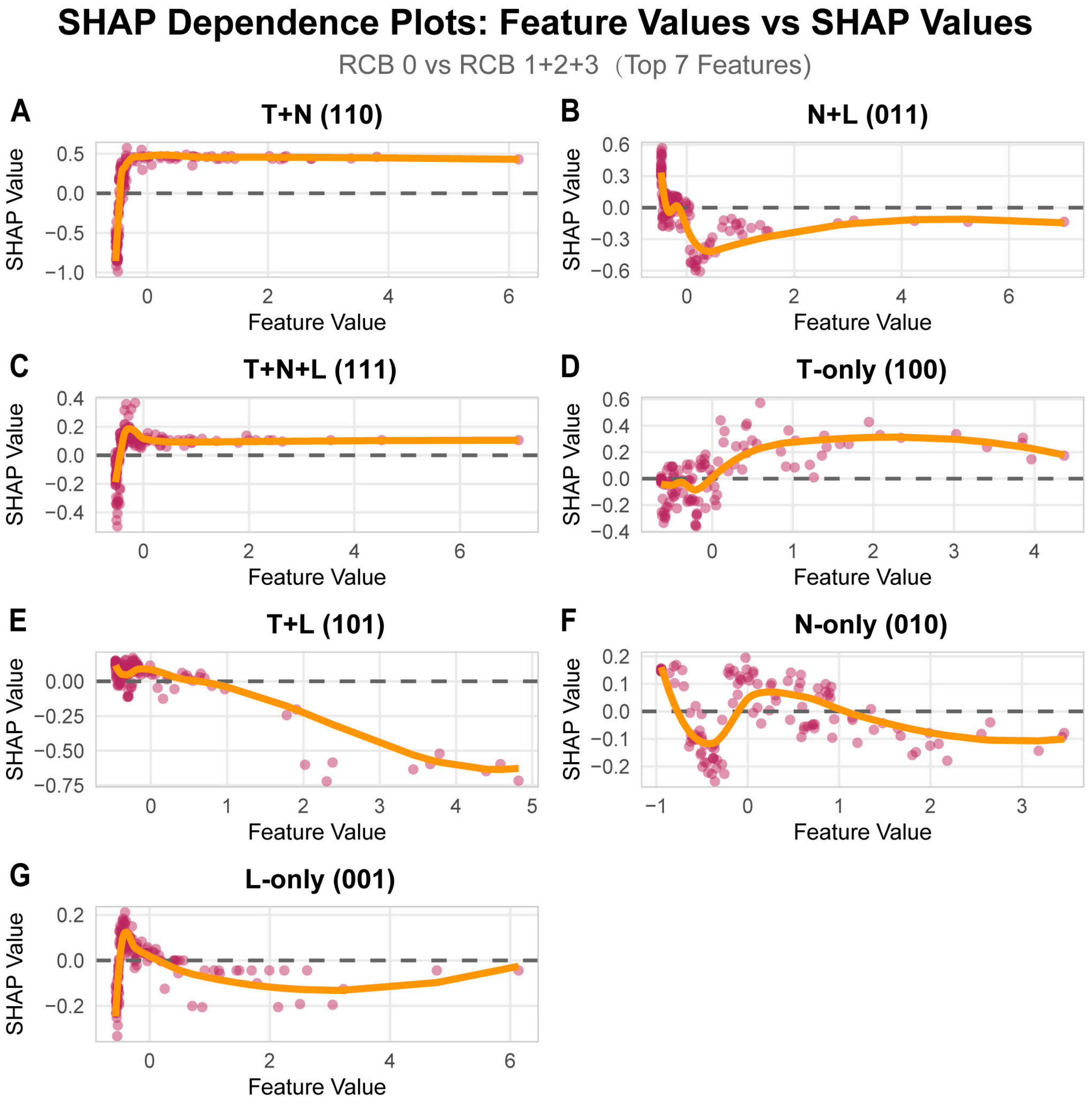

Figure S5. SHAP dependence plots of core features in Task 1 (RCB 0 vs RCB 1+2+3). SHAP dependence plots illustrate nonlinear relationships between values of key combined pathological features and corresponding SHAP values in Task 1, reflecting the direction and magnitude of each feature's effect on model assessment of pathologic complete response (pCR) and non-pathologic complete response (non-pCR). **A.** Necrotic-Tumor Mixed Cluster (T+N, 110). **B.** Lymphocyte-Infiltrated Necrotic Zone (N+L, 011). **C.** Triple-Coexistence Zone (T+N+L, 111). **D.** Pure Tumor-Rich Zone (T-only, 100). **E.** Tumor-Lymphocyte Coexistence (T+L, 101). **F.** Pure Necrotic-Rich Zone (N-only, 010). **G.** Pure Lymphoid-Rich Zone (L-only, 001). Note: Positive SHAP values drive assessment toward RCB 1+2+3 (non-pCR), while negative SHAP values drive assessment toward RCB 0 (pCR).

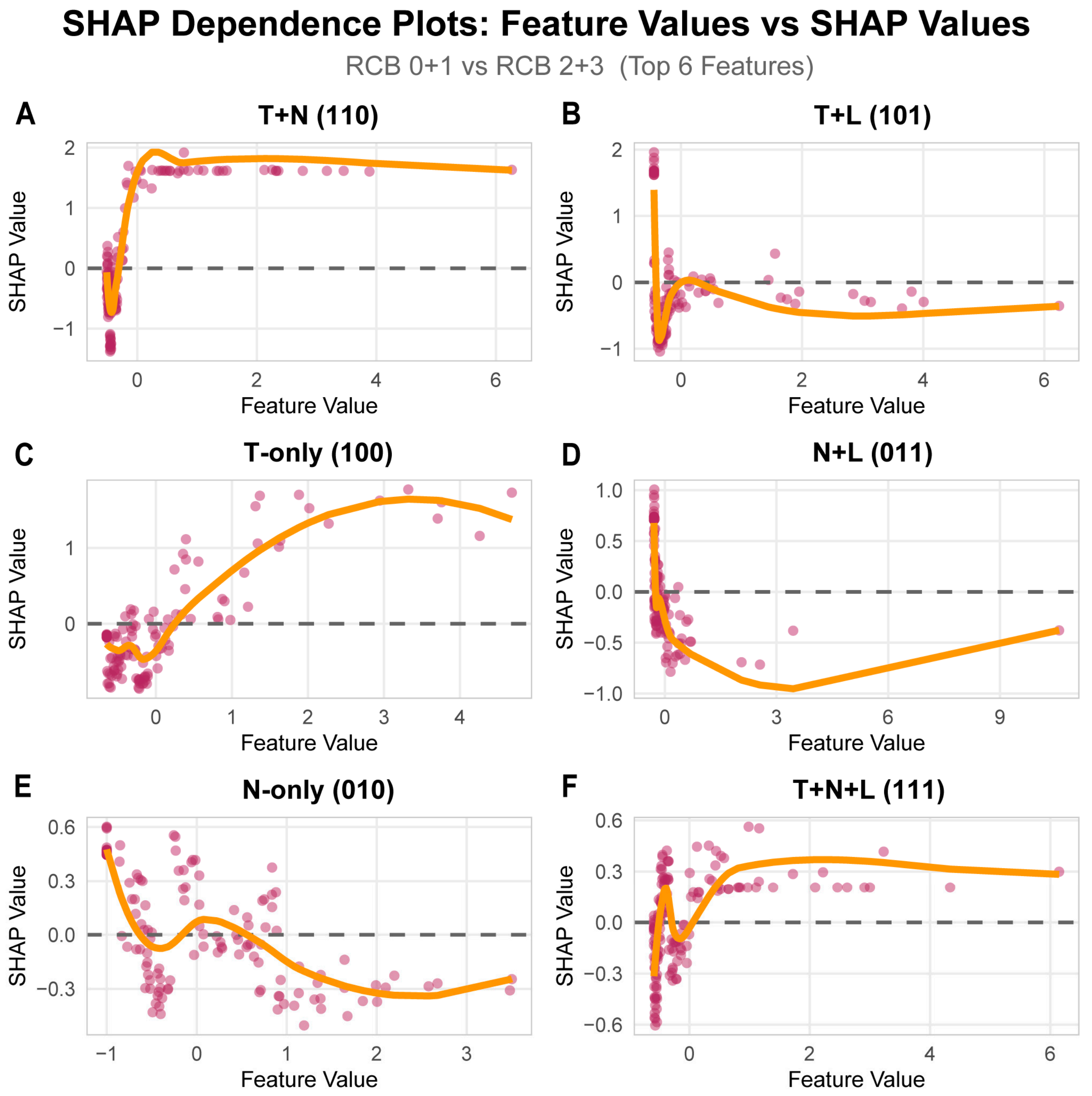

Figure S6. SHAP dependence plots of core features in Task 2 (RCB 0+1 vs RCB 2+3). SHAP dependence plots show nonlinear associations between values of key combined pathological features and SHAP values in Task 2, revealing the contribution patterns of each feature in distinguishing low-intermediate residual cancer burden (RCB 0+1) from high residual cancer burden (RCB 2+3). **A.** Necrotic-Tumor Mixed Cluster (T+N, 110). **B.** Tumor-Lymphocyte Coexistence (T+L, 101). **C.** Pure Tumor-Rich Zone (T-only, 100). **D.** Lymphocyte-Infiltrated Necrotic Zone (N+L, 011). **E.** Pure Necrotic-Rich Zone (N-only, 010). **F.** Triple-Coexistence Zone (T+N+L, 111). Note: Positive SHAP values drive assessment toward RCB 2+3 (high residual burden), while negative SHAP values drive assessment toward RCB 0+1 (low-intermediate residual burden).

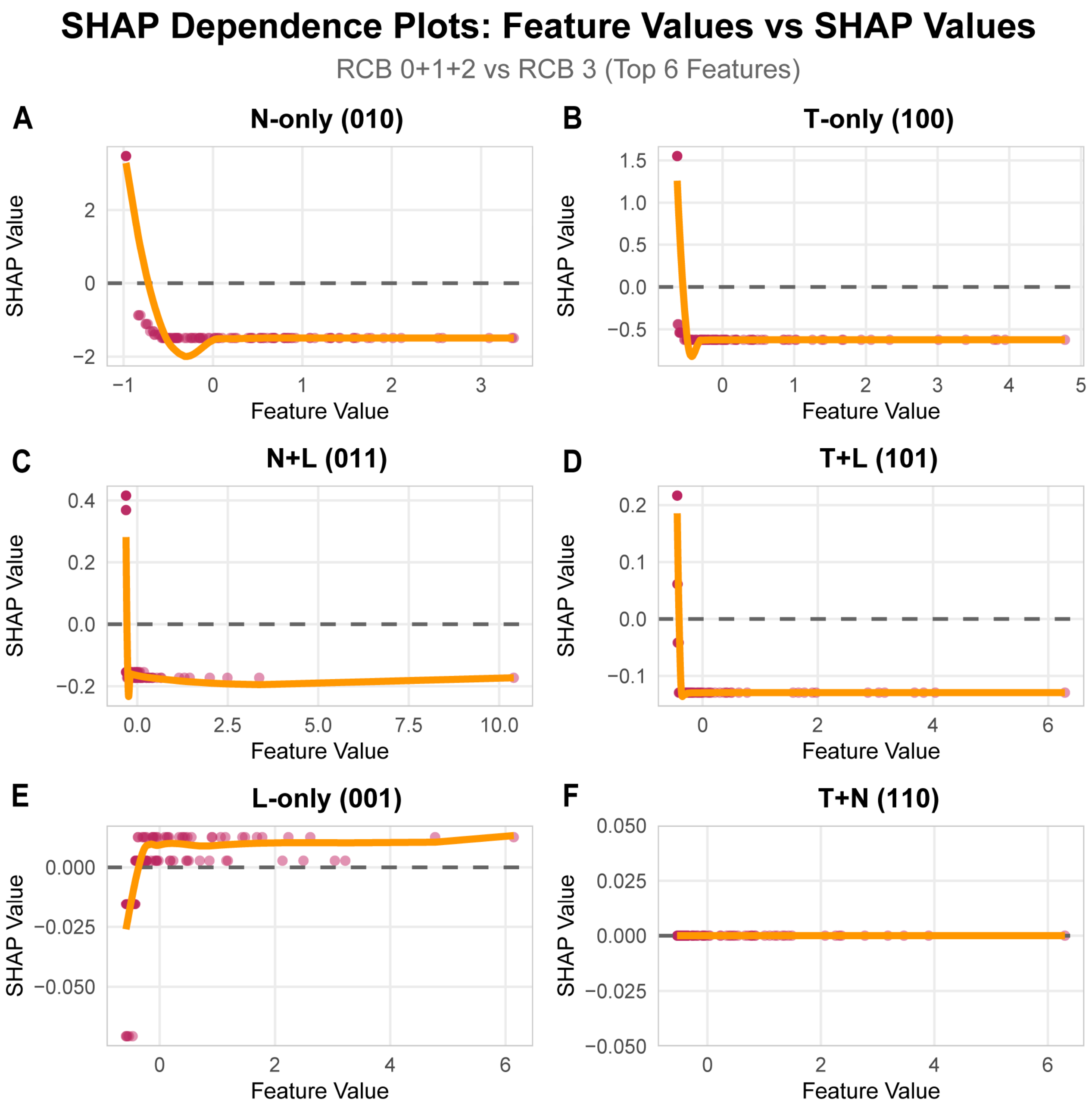

Figure S7. SHAP dependence plots of core features in Task 3 (RCB 0+1+2 vs RCB 3). SHAP dependence plots depict nonlinear relationships between values of key combined pathological features and SHAP values in Task 3, clarifying the effect characteristics of each feature in differentiating low-intermediate residual cancer burden (RCB 0+1+2) from maximal residual cancer burden (RCB 3). **A.** Pure Necrotic-Rich Zone (N-only, 010). **B.** Pure Tumor-Rich Zone (T-only, 100). **C.** Lymphocyte-Infiltrated Necrotic Zone (N+L, 011). **D.** Tumor-Lymphocyte Coexistence (T+L, 101). **E.** Pure Lymphoid-Rich Zone (L-only, 001). **F.** Necrotic-Tumor Mixed Cluster (T+N, 110). Note: Positive SHAP values drive assessment toward RCB 3 (maximal residual burden), while negative SHAP values drive assessment toward RCB 0+1+2 (low-intermediate residual burden).

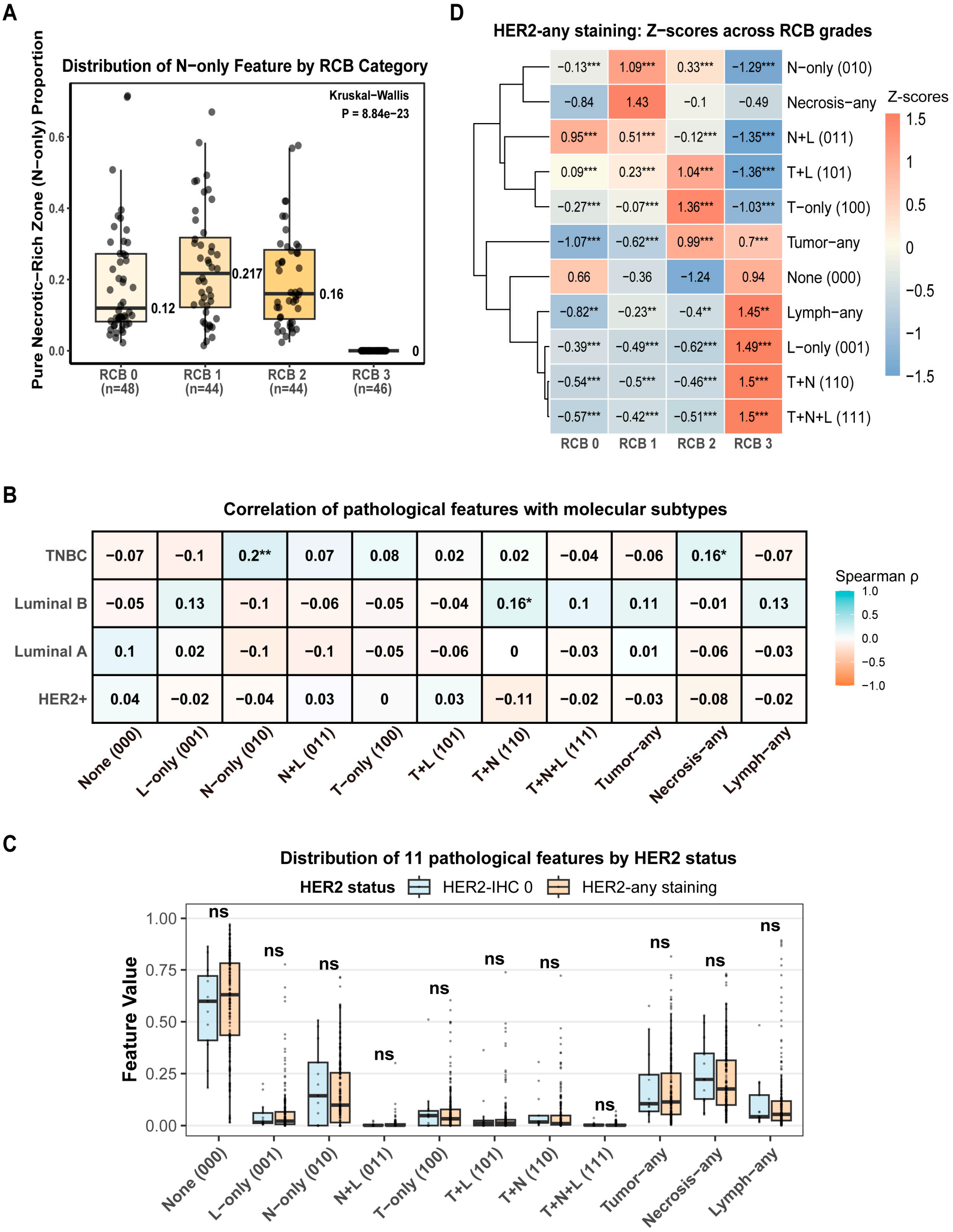

Panel D values (rows: features; columns: RCB 0, RCB 1, RCB 2, RCB 3):

	RCB 0	RCB 1	RCB 2	RCB 3
N−only (010)	−0.13***	1.09***	0.33***	−1.29***
Necrosis−any	−0.84	1.43	−0.1	−0.49
N+L (011)	0.95***	0.51***	−0.12***	−1.35***
T+L (101)	0.09***	0.23***	1.04***	−1.36***
T−only (100)	−0.27***	−0.07***	1.36***	−1.03***
Tumor−any	−1.07***	−0.62***	0.99***	0.7***
None (000)	0.66	−0.36	−1.24	0.94
Lymph−any	−0.82**	−0.23**	−0.4**	1.45**
L−only (001)	−0.39***	−0.49***	−0.62***	1.49***
T+N (110)	−0.54***	−0.5***	−0.46***	1.5***
T+N+L (111)	−0.57***	−0.42***	−0.51***	1.5***

Panel B values (Spearman ρ):

	None (000)	L−only (001)	N−only (010)	N+L (011)	T−only (100)	T+L (101)	T+N (110)	T+N+L (111)	Tumor−any	Necrosis−any	Lymph−any
TNBC	−0.07	−0.1	0.2**	0.07	0.08	0.02	0.02	−0.04	−0.06	0.16*	−0.07
Luminal B	−0.05	0.13	−0.1	−0.06	−0.05	−0.04	0.16*	0.1	0.11	−0.01	0.13
Luminal A	0.1	0.02	−0.1	−0.1	−0.05	−0.06	0	−0.03	0.01	−0.06	−0.03
HER2+	0.04	−0.02	−0.04	0.03	0	0.03	−0.11	−0.02	−0.03	−0.08	−0.02

Figure S8. **A.** Distribution of the Pure Necrotic-Rich Zone (N-only) feature across RCB subgroups in the internal cohort (N = 182). **B.** Heatmap of Spearman correlation coefficients between 11 spatial pathological features and four molecular subtypes (HER2-any staining, Luminal A, Luminal B, and TNBC) in the internal cohort (N = 182). Each cell represents the correlation coefficient (ρ), with red indicating positive correlation and blue indicating negative correlation. Asterisks denote statistical significance. **C.** Boxplots comparing the distribution of 11 pathological features between HER2-any staining and HER2-IHC 0 subgroups in the internal cohort (N = 182). *P* values from Mann-Whitney U tests are shown; no significant differences were observed (all $P > 0.05$). **D.** Heatmap of Z-score normalized median expression of 11 pathological features across RCB grades (0, 1, 2, 3) within the HER2-any staining subgroup (n = 169) of the internal cohort. Asterisks indicate significance from Kruskal-Wallis tests (*** $P < 0.001$, ** $P < 0.01$, * $P < 0.05$).

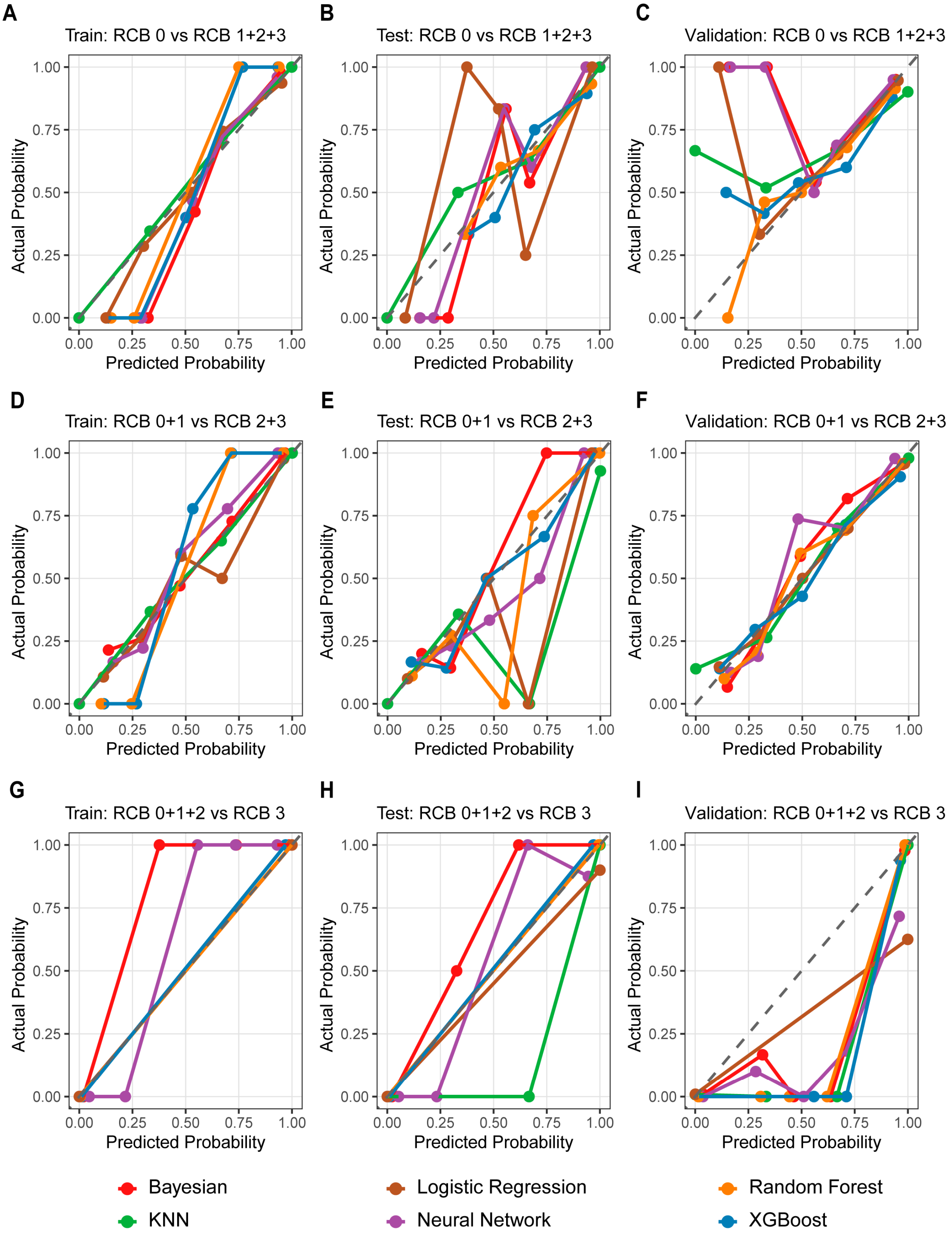
A
Train: RCB 0 vs RCB 1+2+3
B
Test: RCB 0 vs RCB 1+2+3
C
Validation: RCB 0 vs RCB 1+2+3
D
Train: RCB 0+1 vs RCB 2+3
E
Test: RCB 0+1 vs RCB 2+3
F
Validation: RCB 0+1 vs RCB 2+3
G
Train: RCB 0+1+2 vs RCB 3
H
Test: RCB 0+1+2 vs RCB 3
I
Validation: RCB 0+1+2 vs RCB 3
Actual Probability
Predicted Probability
0.00
0.25
0.50
0.75
1.00
Bayesian
KNN
Logistic Regression
Neural Network
Random Forest
XGBoost

Figure S9. Calibration curves of six machine learning models in training, internal test and external validation datasets. **A-C**: Task 1 (RCB 0 vs RCB 1+2+3). **D-F**: Task 2 (RCB 0+1 vs RCB 2+3). **G-I**: Task 3 (RCB 0+1+2 vs RCB 3).

Supplementary Table

Table S1. AUC values with 95% CIs for the six machine learning models in the three tasks.

Model	AUC	CI Lower	CI Upper	Task	Data set
Bayesian	0.823	0.754	0.887	RCB 0 vs RCB 1+2+3	Train
KNN	0.887	0.838	0.928	RCB 0 vs RCB 1+2+3	Train
Logistic Regression	0.836	0.757	0.892	RCB 0 vs RCB 1+2+3	Train
Neural Network	0.816	0.742	0.880	RCB 0 vs RCB 1+2+3	Train
Random Forest	1.000	1.000	1.000	RCB 0 vs RCB 1+2+3	Train
XGBoost	1.000	1.000	1.000	RCB 0 vs RCB 1+2+3	Train
Bayesian	0.795	0.645	0.927	RCB 0 vs RCB 1+2+3	Test
KNN	0.812	0.692	0.923	RCB 0 vs RCB 1+2+3	Test
Logistic Regression	0.812	0.649	0.936	RCB 0 vs RCB 1+2+3	Test
Neural Network	0.791	0.628	0.915	RCB 0 vs RCB 1+2+3	Test
Random Forest	0.763	0.560	0.923	RCB 0 vs RCB 1+2+3	Test
XGBoost	0.833	0.688	0.957	RCB 0 vs RCB 1+2+3	Test
Bayesian	0.768	0.692	0.835	RCB 0 vs RCB 1+2+3	External
KNN	0.712	0.631	0.791	RCB 0 vs RCB 1+2+3	External
Logistic Regression	0.784	0.706	0.850	RCB 0 vs RCB 1+2+3	External
Neural Network	0.772	0.695	0.844	RCB 0 vs RCB 1+2+3	External
Random Forest	0.777	0.699	0.845	RCB 0 vs RCB 1+2+3	External
XGBoost	0.781	0.705	0.845	RCB 0 vs RCB 1+2+3	External
Bayesian	0.856	0.791	0.912	RCB 0+1 vs RCB 2+3	Train
KNN	0.915	0.874	0.950	RCB 0+1 vs RCB 2+3	Train
Logistic Regression	0.856	0.788	0.917	RCB 0+1 vs RCB 2+3	Train
Neural Network	0.875	0.811	0.927	RCB 0+1 vs RCB 2+3	Train
Random Forest	1.000	1.000	1.000	RCB 0+1 vs RCB 2+3	Train
XGBoost	1.000	1.000	1.000	RCB 0+1 vs RCB 2+3	Train
Bayesian	0.886	0.744	0.994	RCB 0+1 vs RCB 2+3	Test
KNN	0.880	0.765	0.966	RCB 0+1 vs RCB 2+3	Test
Logistic Regression	0.892	0.768	0.988	RCB 0+1 vs RCB 2+3	Test
Neural Network	0.880	0.747	0.975	RCB 0+1 vs RCB 2+3	Test
Random Forest	0.903	0.793	0.988	RCB 0+1 vs RCB 2+3	Test
XGBoost	0.895	0.744	0.994	RCB 0+1 vs RCB 2+3	Test
Bayesian	0.869	0.811	0.918	RCB 0+1 vs RCB 2+3	External
KNN	0.877	0.819	0.923	RCB 0+1 vs RCB 2+3	External
Logistic Regression	0.871	0.815	0.923	RCB 0+1 vs RCB 2+3	External
Neural Network	0.885	0.831	0.934	RCB 0+1 vs RCB 2+3	External
Random Forest	0.895	0.838	0.939	RCB 0+1 vs RCB 2+3	External
XGBoost	0.896	0.849	0.937	RCB 0+1 vs RCB 2+3	External
Bayesian	1.000	1.000	1.000	RCB 0+1+2 vs RCB 3	Train
KNN	1.000	1.000	1.000	RCB 0+1+2 vs RCB 3	Train
Logistic Regression	1.000	1.000	1.000	RCB 0+1+2 vs RCB 3	Train
Neural Network	1.000	1.000	1.000	RCB 0+1+2 vs RCB 3	Train
Random Forest	1.000	1.000	1.000	RCB 0+1+2 vs RCB 3	Train
XGBoost	1.000	1.000	1.000	RCB 0+1+2 vs RCB 3	Train
Bayesian	1.000	1.000	1.000	RCB 0+1+2 vs RCB 3	Test

KNN	1.000	1.000	1.000	RCB 0+1+2 vs RCB 3	Test
Logistic Regression	0.979	0.938	1.000	RCB 0+1+2 vs RCB 3	Test
Neural Network	0.984	0.942	1.000	RCB 0+1+2 vs RCB 3	Test
Random Forest	1.000	1.000	1.000	RCB 0+1+2 vs RCB 3	Test
XGBoost	1.000	1.000	1.000	RCB 0+1+2 vs RCB 3	Test
Bayesian	0.989	0.976	0.999	RCB 0+1+2 vs RCB 3	External
KNN	0.988	0.962	1.000	RCB 0+1+2 vs RCB 3	External
Logistic Regression	0.918	0.886	0.950	RCB 0+1+2 vs RCB 3	External
Neural Network	0.979	0.960	0.993	RCB 0+1+2 vs RCB 3	External
Random Forest	1.000	1.000	1.000	RCB 0+1+2 vs RCB 3	External
XGBoost	1.000	1.000	1.000	RCB 0+1+2 vs RCB 3	External

Note. 95% CIs: non-parametric percentile-based bootstrap with 1,000 resamples. CI = [1.000, 1.000] indicates complete separation.

Table S2. Spearman rank correlation between 11 spatial pathological features and model-estimated RCB-like scores in the TCGA-BRCA cohort (exploratory analysis)

**Feature**	**Spearman rho**	**P-value**	**Sample size**
None	-0.285	3.670×10^{-7}	843
Pure Lymphoid-Rich Zone (L-only)	-0.616	4.688×10^{-89}	843
Pure Necrotic-Rich Zone (N-only)	-0.227	2.619×10^{-11}	843
Lymphocyte-Infiltrated Necrotic Zone (N+L)	-0.407	4.927×10^{-35}	843
Pure Tumor-Rich Zone (T-only)	0.706	2.677×10^{-128}	843
Tumor-Lymphocyte Coexistence (T+L)	-0.400	9.608×10^{-34}	843
Necrotic-Tumor Mixed Cluster (T+N)	0.170	6.854×10^{-7}	843
Triple-Coexistence Zone (T+N+L)	-0.192	1.768×10^{-8}	843
Global Tumor Compartment (T-any)	0.558	2.915×10^{-70}	843
Global Necrotic Compartment (N-any)	-0.150	1.213×10^{-5}	843
Global Lymphoid Compartment (L-any)	-0.486	4.222×10^{-51}	843
